# Reirradiation versus systemic therapy versus combination therapy for recurrent high-grade glioma: a systematic review and meta-analysis of survival and toxicity

**DOI:** 10.1007/s11060-023-04441-0

**Published:** 2023-09-21

**Authors:** Ravi Marwah, Daniel Xing, Timothy Squire, Yu Yang Soon, Hui K. Gan, Sweet Ping Ng

**Affiliations:** 1 Department of Radiation Oncology, Townsville University Hospital, 100 Angus Smith Drive, Douglas, Townsville, QLD 4814 Australia; 2 College of Medicine and Dentistry, James Cook University, Townsville, Australia; 3 Department of Radiation Oncology, National University Cancer Institute, Singapore, Singapore; 4 Department of Medical Oncology, Olivia Newton-John Cancer Wellness & Research Centre, Austin Health, Melbourne, Australia; 5 Cancer Therapies and Biology Group, Centre of Research Excellence in Brain Tumours, Olivia Newton-John Cancer Wellness & Research Centre, Austin Hospital, Melbourne, Australia; 6 Department of Radiation Oncology, Olivia Newton-John Cancer Wellness & Research Centre, Austin Health, Melbourne, Australia

**Keywords:** Recurrent, High-grade glioma, Reirradiation, Systemic therapy, Combination therapy

## Abstract

**Purpose:**

This review compares reirradiation (reRT), systemic therapy and combination therapy (reRT & systemic therapy) with regards to overall survival (OS), progression-free survival (PFS), adverse effects (AEs) and quality of life (QoL) in patients with recurrent high-grade glioma (rHGG).

**Methods:**

A search was performed on PubMed, Scopus, Embase and CENTRAL. Studies reporting OS, PFS, AEs and/or QoL and encompassing the following groups were included; reirradiation vs systemic therapy, combination therapy vs systemic therapy, combination therapy vs reRT, and bevacizumab-based combination therapy vs reRT with/without non-bevacizumab-based systemic therapy. Meta-analyses were performed utilising a random effects model. Certainty of evidence was assessed using GRADE.

**Results:**

Thirty-one studies (three randomised, twenty-eight non-randomised) comprising 2084 participants were included. In the combination therapy vs systemic therapy group, combination therapy improved PFS (HR 0.57 (95% CI 0.41–0.79); low certainty) and OS (HR 0.73 (95% CI 0.56–0.95); low certainty) and there was no difference in grade 3 + AEs (RR 1.03 (95% CI 0.57–1.86); very low certainty). In the combination therapy vs reRT group, combination therapy improved PFS (HR 0.52 (95% CI 0.38–0.72); low certainty) and OS (HR 0.69 (95% CI 0.52–0.93); low certainty). In the bevacizumab-based combination therapy vs reRT with/without non-bevacizumab-based systemic therapy group, adding bevacizumab improved PFS (HR 0.46 (95% CI 0.27–0.77); low certainty) and OS (HR 0.42 (95% CI 0.24–0.72; low certainty) and reduced radionecrosis (RR 0.17 (95% CI 0.06–0.48); low certainty).

**Conclusions:**

Combination therapy may improve OS and PFS with acceptable toxicities in patients with rHGG compared to reRT or systemic therapy alone. Particularly, combining bevacizumab with reRT prophylactically reduces radionecrosis.

*Registration*: CRD42022291741.

**Supplementary Information:**

The online version contains supplementary material available at 10.1007/s11060-023-04441-0.

## Introduction

High-grade gliomas (HGG) consist of glioblastoma multiforme (GBM) and anaplastic gliomas (anaplastic astrocytoma and anaplastic oligodendroglioma) [1]. Most HGGs are managed with a multimodal approach, incorporating maximal safe surgical resection, postoperative radiotherapy and temozolomide [2]. Despite this, HGGs have a poor prognosis with a median overall survival (OS) of 15 months and nearly all patients experiencing tumour recurrence [3]. Treatment options for recurrent HGG (rHGG) are limited and include reoperation, reirradiation (reRT), second-line chemotherapy, or a combination of these [4].

Notably, 90% of recurrences occur within 2 cm of the original tumour site, suggesting the need for improved local control [5]. To control local progression, surgical resection is one treatment option, with a median reported OS of 9.7 months. However, only 20–30% of patients with recurrent or progressive disease have resectable lesions, and reoperation is often limited by performance status and diffuse, infiltrative disease involving eloquent areas [4].

ReRT is another localised treatment option for recurrent or progressive disease. It generally benefits patients with a good performance status (KPS > 60), localised/unifocal disease, and a time interval between initial radiation and reRT of at least 6 months [6, 7]. Retrospective data suggests that reRT is safe and provides improved local control [8], with a median reported OS of 7.5–16 months [6] and an OS-12 rate of 36% in recurrent GBM (rGBM) [9]. ReRT is controversial given the tendency for in/near-field recurrences, and hence the increased risk for radiation necrosis (RN) from cumulative dose. The reported incidence of RN following reRT is 0–31.3% [10]. Risk factors associated with RN include fractionation schedule, dose (cumulative EQD2), treatment volume, time interval between initial radiation and reRT, and concomitant systemic therapy. Three external beam radiotherapy techniques are used based on fractionation schedule; stereotactic radiosurgery (SRS), hypofractionated stereotactic radiotherapy (HFSRT), and conventionally fractionated radiotherapy (conventional RT) [7].

Second-line chemotherapy is commonly required for durable tumour control. Literature reports a median OS of 6–9 months in patients with rGBM treated with salvage chemotherapy. No single agent or combination of systemic therapies has demonstrated superiority to the others [11–13]. Bevacizumab has demonstrated efficacy in delaying tumour progression, albeit without an OS improvement, and hence has been approved by the FDA for treatment of rGBM [14]. Nevertheless, most patients with rHGG progress on bevacizumab after a median time of 3–5 months [15].

Several retrospective studies report the safety and efficacy of combining reRT with bevacizumab [16]. In a network meta-analysis of treatment options for progressive or rGBM, McBain et al. found limited evidence suggesting reRT with or without bevacizumab may improve survival in select individuals [17]. Bevacizumab is further postulated to reduce radioresistance and to lower the incidence of RN [18]. Temozolomide has also been studied in combination with reRT in rHGG due to its radio-sensitizing effect [19]. However, its value in this setting is uncertain given its widespread use in initial treatment and possible subsequent resistance [20].

Combination therapy with localised treatment and systemic therapy allows for the simultaneous targeting of macroscopic, microscopic, and diffuse disease, and hence may result in improvements in OS and progression-free survival (PFS). However, as the treatment of rHGG is of palliative intent, cumulative treatment-related toxicities must be balanced with the potential impact of disease progression on quality of life (QoL). This review aims to compare the impact of reRT, systemic therapy and combination therapy (reRT & systemic therapy) with regards to OS, PFS, adverse effects (AEs), and quality of life (QoL) in patients with rHGG.

## Methods

This review was conducted in accordance with PRISMA guidelines.

### Search strategy

A search was performed on PubMed, Scopus, Embase and CENTRAL on 18 March 2022. A repeat search was conducted on 1 February 2023. The search was limited to studies published from 2010, with no limits on language.

The following Boolean search was utilised;

(“radiation” OR “radiotherapy” OR “radio-therapy” OR “radio therapy” OR “irradiation” OR “reirradiation” OR “re-irradiation” OR “re irradiation” OR “RT” OR “stereotactic” OR “radiosurgery” OR “radio-surgery” OR “radio surgery”) AND (“glioblastoma” OR “GBM” OR “high grade glioma” OR “high grade gliomas” OR “high-grade glioma” OR “high-grade gliomas” OR “malignant glioma” OR “malignant gliomas”) AND (“recurrent” OR “recurrence” OR “progressive”).

### Eligibility criteria

Studies reporting OS, PFS, AEs and/or QoL in patients with rHGG, and encompassing the following groups were included; reirradiation vs systemic therapy, combination therapy vs systemic therapy, combination therapy vs reirradiation, and bevacizumab-based combination therapy vs reirradiation ± non-bevacizumab-based systemic therapy. Studies were included only if patients received external beam radiotherapy.

Studies were excluded if they were single-arm studies, reviews, case reports, conference abstracts, animal studies or in-vitro studies, or if they did not strictly encompass the comparative treatment groups or report any outcomes of interest. Studies incorporating patients receiving brachytherapy and patients with low grade gliomas or other non-glial CNS tumours were also excluded.

The first author (R.M) independently conducted title/abstract screening. Both first and second authors (R.M and D.X) performed full text screening individually. Disagreements were resolved by consensus. The reference lists of included manuscripts were examined to identify additional articles.

### Data collection

Data was extracted on study characteristics, demographics, tumour characteristics, intervention characteristics, and outcome measures of interest (OS, PFS, AEs (RN (any grade), CTCAE Grade 3 + toxicities, treatment-related deaths), QoL) by the first author (R.M) and cross-checked by the second author (D.X). To maintain consistent definitions, OS and PFS were only collected for studies which measured these outcomes from recurrence or retreatment.

### Quality assessment

Risk of bias (RoB) was independently assessed by first and second authors (R.M & D.X) using the Cochrane RoB 2 tool [21] for randomised control trials (RCTs) and the ROBINS-I tool [22] for non-randomised studies. Differences between authors were resolved with discussion on completion. Publication bias was assessed through the visual inspection of funnel plots generated using the RevMan 5.4 software.

### Synthesis methods

Only studies of low to moderate RoB were included in the meta-analyses, while studies of serious or critical RoB were excluded. Meta-analyses were performed for outcome measures of interest utilising the RevMan 5.4 software. Meta-analyses could not be conducted for QoL due to insufficient reporting. The logHR and SE(logHR) for OS and PFS, and logRR and SE(logRR) for AEs were extracted or estimated if not reported [23]. Data was pooled by each comparative treatment group using the generic inverse variance method, and the DerSimionian and Laird random effects model was utilised given expected heterogeneity. A forest plot was generated for each outcome measure within each comparative treatment group. Statistical significance was defined as p < 0.05 and 95% confidence intervals were reported. A subset analysis was performed for only RCTs if ≥ 2 studies were available. Further subset analysis was also performed for GBM-only studies.

### GRADE approach

The overall certainty of evidence was assessed for each outcome using the Grading, Recommendations, Assessment, Development and Evaluation (GRADE) approach [24].

## Results

### Study selection

**Fig. 1:**
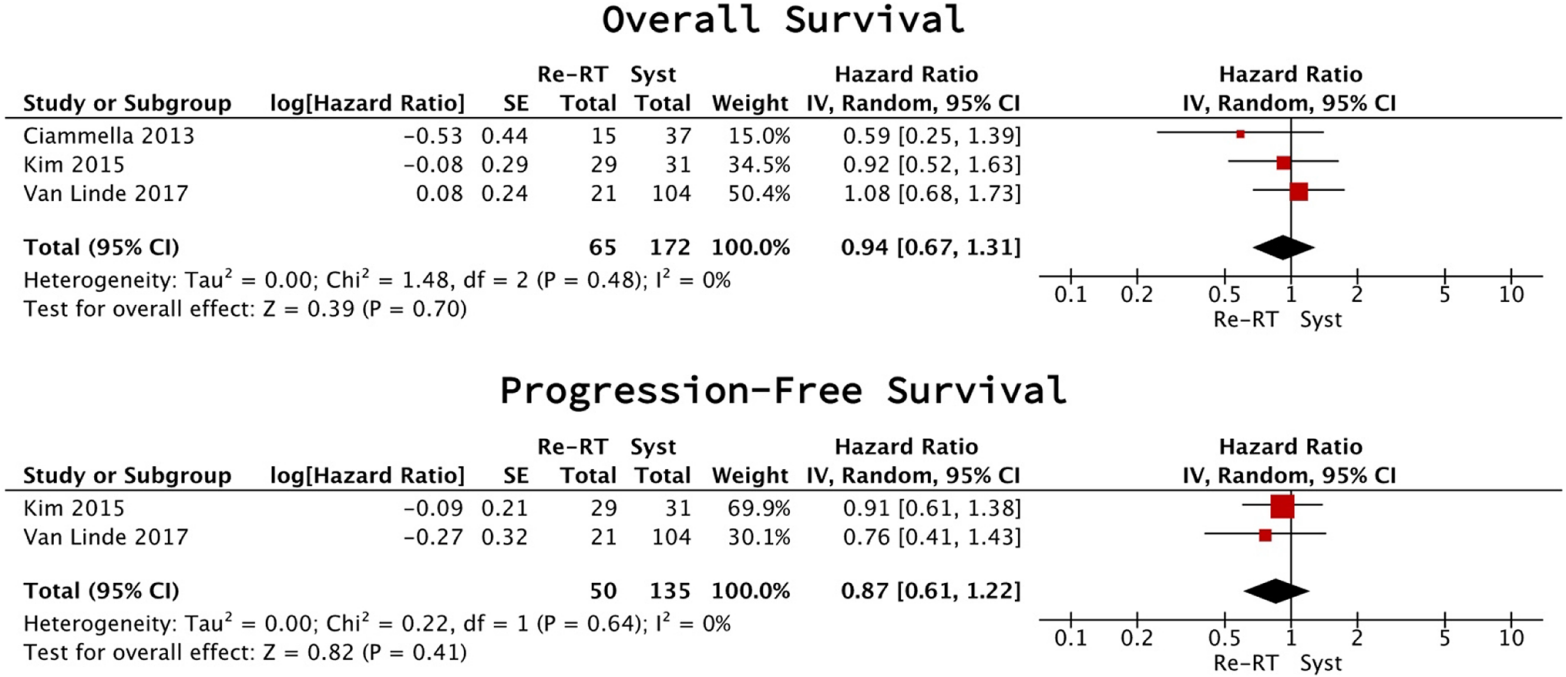
Reirradiation vs systemic therapy meta-analysis (rHGG)

As demonstrated in Supplementary Material; Fig. 1, 12,244 studies were identified in the initial search, with 7338 studies remaining after removing duplicates. 7279 studies were excluded on title and abstract screening resulting in 59 articles for full-text screen. Of these articles, 28 were included; 9 were excluded as they were non-comparative, 19 were excluded as they did not strictly encompass the comparative treatment groups, 2 were excluded as they included patients treated with brachytherapy, and 1 was excluded as it included patients with low grade gliomas or other non-glial CNS tumours. 3 additional articles [25–27] were included after an updated search was conducted on 1 February 2023, resulting in a total of 31 articles. No additional articles were included on examination of reference lists of included studies.

### Study and treatment characteristics

Study and treatment characteristics are summarised in Tables 1 and 2. Thirty-one studies (three RCTs, one matched-case control study, twenty-seven cohort studies) comprising 2084 participants were included. Participants incorporated 1076 males, 739 females, and 269 individuals of unspecified sex. 1593 participants had WHO Grade IV tumours, 210 had WHO Grade III tumours, and 281 had non-specified HGGs. 4 studies [28–31] comprised the reirradiation vs systemic therapy group, 7 studies [26, 27, 31–35] encompassed the combination therapy vs systemic therapy group, 17 studies [31, 36–51] comprised the combination therapy vs reirradiation group, and 8 studies [18, 25, 49–54] encompassed the bevacizumab-based combination therapy vs reirradiation with/without non-bevacizumab-based systemic therapy group.

**Table 1:** Study characteristics

Study	Group	Tumour type	Study design	Sample size	Country	WHO Grade	IDH	MGMT	Age (years)	Sex	KPS	Initial treatment	Initial XRT
Van Linde [28]	Syst vs reRT	rGBM	Cohort study	125 (syst = 104, reRT = 21)	Netherlands	Syst; Grade IV = 104 reRT; Grade IV = 21	NR	NR	Syst = 59 (19–77) reRT = 59 (26–71)	Syst = 71 M, 33F reRT = 10 M, 11F	Syst = range; 40–100 reRT = range; 50–100	Resection + ChemoRT	NR
Ciammella [29]	Syst vs reRT	rGBM	Cohort study	52 (syst = 37, reRT = 15)	Italy	Syst; Grade IV = 37 reRT; Grade IV = 15	NR	Syst = 12/32 reRT = 7/12	Syst = 66 (49–72) reRT = 51.5 (41–73)	Syst = 22 M, 15F reRT = 11 m, 4F	Syst = 80 (70–100) reRT = 90 (80–100) criteria; KPS >/60	Maximal resection + ChemoRT (Stupp Protocol)	60 Gy/30#
Socha [30]	Syst vs reRT	rGBM	Prospective cohort study	24 (syst = 21, reRT = 3)	Belarus, Brazil, Chile, Georgia, Greece, India, Indonesia, Ireland, Poland, Thailand, Tunisia	Syst; Grade IV = 21 reRT; Grade IV = 3	NR	NR	Criteria: >/50 years	NR	NR	Maximal resection + HF-SRT	25 Gy/5# or 40 Gy/15#
Kim [31]	Syst vs comb reRT vs comb	rGBM	Cohort study	59 (syst = 31, comb = 28) 57 (reRT 29, comb = 28)	Korea	Syst; Grade IV = 31 reRT; Grade IV = 29 comb; Grade IV = 28	NR	NR	Syst = 50 (30–68) reRT = 61 (35–87) comb = 53 (28–73)	Syst = 19 M, 12F reRT = 13 M, 16F comb = 16 M, 12F	NR	Maximal resection + concurrent ChemoRT (TMZ)	50–60 Gy with conventional fractionation
Bovi [32]	Syst vs comb	rHGG	Cohort study	80 (syst = 47, comb = 33)	USA	Syst; Grade III = 12 Grade IV = 35 comb; Grade III = 14 Grade IV = 19	NR	NR	Syst = 60 (23–79) comb = 42 (26–71)	Syst = 28 M, 19F comb = 19 M, 14F	Syst = 70 (40–100) comb = 80 (60–100)	NR	NR
Bergman [33]	Syst vs comb	rHGG	RCT	35 (syst = 17, comb 18)	USA	Grade III = 6 Grade IV = 29	Syst; 1/12 comb; 1/14	Syst = 4/13 comb = 7/13	Syst = 59 (39–74) comb = 53 (27–81)	syst = 11 M, 6F comb = 14 M, 4F	Syst = 80 (70–100) comb = 80 (70–90) (criteria; KPS > /70)	Resection + radiation ± TMZ	60 Gy/30#
Yasuda [34]	Syst vs comb	rHGG	Cohort study	58 (syst = 29, comb = 29)	Japan	SYST; Grade III = 7 Grade IV = 22 comb; Grade III = 13 Grade IV = 16	Syst; 9/27 comb;10/27	NR	Syst = 21 (< 50 years), 8 (> / = 50 years) comb = 10 (< 50 years), 19 (> / = 50 years)	Syst = 19 M, 10F comb = 15 M, 14F	80 (50–100)	Resection + radiation ± chemotherapy	60 Gy with conventional fractionation
Schnell [35]	Syst vs comb	rHGG	Cohort study	Syst vs comb (concurrent-maintenance); 58 (syst = 30, comb = 28) syst vs comb (concurrent); 77 (syst = 30, comb = 47)	Germany	Syst; Grade III = 3 Grade IV = 27 comb; Grade III = 17 Grade IV = 58	Syst; 6/28 comb; 13/68	Syst; 14/28 comb; 37/68	Syst = 48.5 comb = 52 Criteria: </75 years	Syst = 18 M, 12F comb = 48 M, 27F	Syst = 70 comb (concurrent) = 80 comb (concurrent = maintenance) = 90	Resection + radiation ± chemotherapy (TMZ)	60 Gy with conventional fractionation
Lee [26]	Syst vs comb	rHGG	Cohort study	203 (syst = 135, comb = 68)	Korea	NR	32/188	89/187	54 (19–76)	115 M, 88F	NR	Maximal resection + ChemoRT (TMZ)—Stupp protocol	NR
Tsien [27]	Syst vs comb	rGBM	RCT; Phase II	170 (syst = 84, comb = 86)	USA (Multi-Institutional)	Syst; Grade IV = 84 comb; Grade IV = 86	NR	Syst = 12/36 comb = 18/45	Syst = 57 (25–87) comb = 60 (28–81)	Syst = 46 M, 38F comb = 43 M, 43F	Syst = 70–80 (60–100) comb = 70–80 (60–100) (criteria; KPS > /60)	NR	NR
Yazici [36]	reRT vs comb	rGBM	Cohort study	37 (reRT = 26, comb = 11)	Turkey	reRT; Grade IV = 26 comb; Grade IV = 11	NR	NR	37 (22–69)	18 M, 19F	Criteria; KPS >/60	Resection + ChemoRT (TMZ)	60 Gy/30#
Wick [37]	reRT vs comb	rGBM	RCT	84 (reRT = 26, comb = 58)	Germany	reRT; Grade IV = 26 comb; Grade IV = 58	reRT = 0/25 comb = 6/55	reRT = 15/23 comb = 41/55	reRT = 59 (25–79) comb = 57 (20–73)	reRT = 12 M, 14F comb = 39 M, 19F	reRT = 90–100 (60–100) comb = 90–100 (60–100) (criteria; KPS > /60)	NR	< 60 Gy
Miwa [38]	reRT vs comb	rGBM	Prospective cohort study	21 (reRT = 8, comb = 13)	Japan	reRT; Grade IV = 8 comb; Grade IV = 13	NR	NR	53.9 (22–76)	18 M, 3F	80 (60–90) Criteria; KPS >/60	Resection + ChemoRT (TMZ)	60 Gy (range 54–68)/30#
Lovo [39]	reRT vs comb	rGBM	Cohort study	46 (reRT = 31, comb = 15)	Spain, El Salvador, Costa Rica	reRT; Grade IV = 31 comb; Grade IV = 15	NR	NR	Mean; 50.3 (19–81)	28 M, 18F	Range: 50–100	Resection + Concurrent ChemorRT (TMZ, TMZ + Bev, BCNU)	NR
Baehr [40]	reRT vs comb	rGBM	Cohort study	40 (reRT = 10, comb = 30)	Germany	reRT; Grade IV = 10 comb; Grade IV = 30	3/25	14/38	57.5 (34–79)	22 M, 18F	NR	Resection + Concurrent ChemoRT (TMZ)	60 Gy/30#
Hasan [41]	reRT vs comb	rGBM	Cohort study	19 (reRT = 3, comb = 16)	USA	reRT; Grade IV = 3 comb; Grade IV = 16	NR	NR	55 (28–78)	13 M, 6F	80 (40–90)	Resection + Concurrent ChemoRT (TMZ)	60 Gy (54–60)/28–32#
Conti [42]	reRT vs comb	rGBM	Prospective cohort study	23 (reRT = 11, comb = 12)	Italy	reRT; Grade IV = 11 comb; Grade IV = 12	NR	NR	Criteria: </70 years 58 (45–70)	10 M, 13F	Criteria; KPS > 70	Resection + ChemoRT (TMZ)-stupps regimen	60 Gy/30#
Fogh [43]	reRT vs comb	rHGG	Cohort study	147 (reRT = 99, comb = 48)	USA	Grade III = 42 Grade IV = 105	NR	NR	53 (19–86)	NR	Criteria; KPS >/60	Resection + radiation ± chemotherapy	60 Gy/30#
Eberle [44]	reRT vs comb	rHGG	Cohort study	30 (reRT = 6, comb = 24)	Germany	Grade III-7 Grade IV-23	3/28	17/29	59 (28–76)	14 M, 16F	70	Radiation with/without resection ± chemotherapy (TMZ)	60 Gy (37.5–61.2)/30#
Saeed [45]	reRT vs comb	rGBM	Prospective cohort study	45 (reRT = 14, comb = 31)	USA	reRT; Grade IV = 14 comb; Grade IV = 31	NR	16/32	54 (27–81)	22 M, 23F	NR	Radiation with/without resection ± chemotherapy	60 Gy (25–60)/30#
Scartoni [46]	reRT vs comb	rGBM	Cohort study	26 (reRT = 19, comb = 7)	Italy	reRT; Grade IV = 19 comb; Grade IV = 7	NR	NR	53.4 (30–69)	18 M, 8F	80 (60–100) (criteria; KPS >/60)	Radiation + concomitant/adjuvant temozolomide	60 Gy/30#
Shen [47]	reRT vs comb	rHGG	Cohort study	118 (reRT = 22, comb = 96)	USA	Grade III = 30 Grade IV = 87 NR = 1	18/52	29/55	47 (14–78)	61 M, 57F	80 (40–100)	Radiation with/without resection	60 Gy (45–60)/30#
Cheon [48]	reRT vs comb	rHGG	Cohort study	29 (reRT = 16, comb = 13)	Korea	NR	NR	NR	53.8 (20–75)	10 M, 19F	NR	Radiation with/without chemotherapy (TMZ/PCV/nimustine + cisplatin/carmustine + cisplatin)	40–59.4 Gy
Park [49]	reRT vs comb reRT vs reRT + Bev	rGBM	Matched case–control study	55 (reRT = 44, comb = 11)	USA	reRT; Grade IV = 44 comb; Grade IV = 11	NR	NR	reRT: 64 (41–77) comb: 62 (46–72)	reRT: 28 M, 16F comb: 8 M, 3F	reRT = 90 (70–100) reRT + Bev = 90 (80–100)	Maximal Resection + ChemoRT (TMZ)-Stupp Protocol	60 Gy (54–60)/30#
Chan [50]	reRT vs comb reRT vs reRT + Bev	rHGG	cohort study	67 (reRT = 6, comb = 61)	Australia	Grade III = 16 Grade IV = 51	17/67	NR	54 (26–83)	43 M, 24F	NR	Definitive radiotherapy or resection with high dose adjuvant radiotherapy	60 Gy (40–60)/30# (15–30)
Hundsberger [51]	reRT vs comb reRT vs reRT + Bev	rHGG	Cohort study	14 (reRT = 4, comb = 10)	USA	Grade III = 6 Grade IV = 8	NR	NR	45 (28–68)	10 M, 4F	70 (70–90)	Radiation with/without resection ± chemotherapy (TMZ)	60/30#
Guan [52]	reRT vs reRT + Bev	rGBM	Cohort study	49 (reRT = 25, reRT + Bev = 24)	China	reRT; Grade IV = 25 reRT + Bev; Grade IV = 24	NR	NR	NR	NR	70 (40–90)	Maximal safe resection and adjuvant radiation treatment + concurrent and maintenance TMZ	60 Gy/30#
Fleischmann [18]	reRT vs reRT + Bev	rHGG	Prospective cohort study	161 (reRT = 37, reRT + Bev = 124)	Germany	reRT; Grade III = 8 Grade IV = 29 reRT + Bev; Grade III = 29 Grade IV = 95	reRT = 9/28 comb = 17/79	reRT = 24/34 comb = 63/117	reRT = 51 (30–75) comb = 50.5 years (18–81)	reRT = 22 M, 15F comb = 85 M, 39F	reRT = 80 (50–100) reRT + Bev = 80 (40–100) Criteria; KPS > 70	Tumour resection + adjuvant radiation ± concomitant and adjuvant TMZ	60 Gy/30# or 40 Gy/15# (for elderly subpopulation > 65 years)
Cuneo [53]	reRT vs reRT + Bev	rGBM	Cohort study	49 (reRT = 16, reRT + Bev = 33)	USA	reRT; Grade IV = 16 reRT + Bev; Grade IV = 33	NR	NR	NR	NR	reRT = 80 reRT + Bev = 80	Gross or near total resection followed by adjuvant radiation and TMZ	60 Gy/30#
Helis [25]	reRT vs reRT + Bev	rGBM	Cohort study	36 (reRT (± chemo) = 22, reRT + Bev (± chemo) = 14	USA	reRT; Grade IV = 22 reRT + Bev; Grade IV = 14	7/36	8/36	NR	26 M, 10F	80 (40–90)	Conventional radiotherapy	NR
Youland [54]	reRT vs reRT + Bev	rHGG	Cohort study	48 (reRT (± chemo) = 21, reRT + Bev (± chemo) = 27)	USA	NR	5/28	9/15	55 (22–72)	31 M, 17F	NR	Resection + radiation ± chemotherapy (TMZ ± experimental agents)	60 Gy (40–76)/30# (15–36)

*Syst* systemic therapy, *ReRT* reirradiation, *Comb* combination therapy, *Bev* bevacizumab, *rGBM* recurrent glioblastoma multiforme, *rHGG* recurrent high-grade glioma, *RCT* randomised control trial, *IDH* isocitrate dehydrogenase, *MGMT* O-6-methylguanine-DNA methyltransferase, *TMZ* temozolomide, *Chemo* chemotherapy, *RT* radiotherapy, *Gy* Gray, *#* fractions, *XRT* radiotherapy, *KPS* Karnofsky Performance Scale, *NR* not reported

**Table 2:** Treatment characteristics & outcomes

Study	Group	Syst type	ReRT type	Comb type	ReRT	EQD2 (a/B = 10)) (Gy)	Planning target volume (cc)
van Linde [28]	Syst vs reRT	Various (Lomustine/lomustine + BEV/TMZ/BEV/PCV/others)	NR	–	NR	NR	NR
Ciammella [29]	Syst vs reRT	NR	HF-SRT	–	25 Gy/5#	31.25 Gy	NR
Socha [30]	Syst vs reRT	TMZ	NR	–	NR	NR	NR
Kim [31]	Syst vs comb reRT vs comb	TMZ	SRS	SRS + concurrent TMZ	15 Gy/1#	31.25	NR
Bovi [32]	Syst vs comb	BEV	–	Pulsed reduced-dose RT + concurrent/concurrent-maintenance BEV	52 Gy/26 sessions	52	NR
Bergman [33]	Syst vs comb	BEV-based Chemo ± irinotecan ± TMZ ± carboplatin ± etoposide	–	HF-SRT + concurrent-maintenance BEV-based Chemo + irinotecan/TMZ/carboplatin/etoposide	32 Gy/4#	48	NR
Yasuda [34]	Syst vs comb	BEV-based chemo	–	HF-SRT + BEV-based chemo	42 Gy/7#	56	33.9 (range, 2.2–305.7)
Schnell [35]	Syst vs comb	BEV + irinotecan	–	Conventional RT + concurrent/concurrent-maintenance BEV	36 Gy/18#	36	ReRT + concomitant BEV = 136.6 ReRT + concomitant/maintenance BEV = 105.8
Lee [26]	Syst vs comb	BEV + irinotecan	–	HF-SRT + adjuvant BEV + irinotecan	24 Gy (12–35)/1–5#	29.6–68	NR
Tsien [27]	Syst vs comb	BEV	–	HF-SRT + concurrent-maintenance BEV	35 Gy/10#	39.38	54 (4–412)
Yazici [36]	reRT vs comb	–	HF-SRT	HF-SRT + adjuvant TMZ/BEV + irinotecan/lomustine	30 Gy (14–32)/5# (1–5)	40	NR
Wick [37]	reRT vs comb	–	Conventional RT	Conventional RT + concurrent-maintenance APG101	36 Gy/18#	36	NR
Miwa [38]	reRT vs comb	–	HF-SRT	HF-SRT + adjuvant TMZ	30 (25–35) Gy/5#	40	Mean PTV = 27.4 ± 24.1 (3.4–102.9)
Lovo [39]	reRT vs comb	–	SRS	SRS + BEV/TMZ	14–16 Gy (8-24 Gy)/1–5#	14.67–31.25	NR
Baehr [40]	reRT vs comb	–	Conventional RT	Conventional RT + concurrent TMZ/BEV/nitrosureas	39.6 Gy (30–50.4)/20#	39.53	120.5 (25–580)
Hasan [41]	reRT vs comb	–	HF-SRT	HF-SRT + adjuvant BEV ± TMZ ± (125)I-mAb 425	25 Gy (18–35)/3–5#	31.25–38.19	NR
Conti [42]	reRT vs comb	–	HF-SRT	HF-SRT + concurrent-maintenance TMZ	20 Gy (15–27.5)/2# (1–5)	33.33	reRT + syst; Mean PTV 13.8 ± 8.3 reRT; 15.1 ± 8.2
Fogh [43]	reRT vs comb	–	HF-SRT	HF-SRT + concurrent TMZ/TMZ + BEV + irinotecan/TMZ + bortezomib/BEV + irinotecan/epothilone/sunitinib/sorafenib/vincristine/carboplatin	35 Gy/10#	39.38	NR
Eberle [44]	reRT vs comb	–	HF-SRT (Carbon-ion)	HF-SRT (carbon–ion) + adjuvant chemotherapy (unspecified)	45 Gy/15#	48.75	NR
Saeed [45]	reRT vs comb	–	HF-SRT (Proton)	HF-SRT (proton) + TMZ/BEV/TMZ + BEV/vorinostat + BEV	46.2 Gy (25–60)/21#	46.97	NR
Scartoni [46]	reRT vs comb	–	Conventional RT (Proton)	Conventional RT (proton) + concurrent TMZ	36 Gy/18#	36	118
Shen [47]	reRT vs comb	–	Conventional RT	Conventional RT + concurrent TMZ/BEV/TMZ + BEV	41.4 Gy (12.6–54)/conventional fractionation	41.4	NR
Cheon, 2018 [48]	reRT vs comb	–	SRS	SRS + concurrent TMZ/PCV/ICE	16 Gy (10–24)	NR	NR
Park, 2012 [49]	reRT vs comb reRT vs reRT + Bev	–	SRS	SRS + adjuvant BEV-based chemo + TMZ/irinotecan	16 Gy (13-18 Gy)/1#	34.67	NR
Chan, 2020 [50]	reRT vs comb reRT vs reRT + Bev	–	HF-SRT	HF-SRT + concurrent-maintenance BEV	35 Gy (35–40)/15# (10–15)	35.97	145.3 (range 10.6–432.8)
Hundsberger [51]	reRT vs comb reRT vs reRT + Bev	–	HF-SRT	HF-SRT + concurrent-maintenance BEV	41.6 Gy (39–55)/2.66 Gy per fraction	43.89	190 (47–373)
Guan [52]	reRT vs reRT + Bev	–	HF-SRT ± concurrent temozolomide	HF-SRT + concurrent BEV-based chemotherapy ± TMZ	24 Gy (12–30 Gy)/4# (2–6#)	32	16.68 (0.81–121.96)
Fleischmann [18]	reRT vs reRT + Bev	–	Conventional RT ± concurrent TMZ	Conventional RT + concurrent/concurrent-maintenance BEV	36 Gy (30–48.4 Gy)/18#	36	reRT; 122.46 (43.39–293.51) reRT + Bev; 117.45 (22.55–385.5)
Cuneo [53]	reRT vs reRT + Bev	–	SRS ± irinotecan ± lomustine ± etoposide	SRS + BEV-based chemo ± irinotecan ± lomustine ± etoposide	15 Gy (12.5–25 Gy)/1# (1 or 5#)	31.25	4.8
Helis [25]	reRT vs reRT + Bev	–	Conventional RT ± TMZ ± other	Conventional RT + BEV-based chemo ± TMZ	NR	NR	214.4
Youland [54]	reRT vs reRT + Bev	–	HF-SRT ± TMZ	HF-SRT + concurrent BEV/BEV + lomustine/BEV + TMZ	35 Gy(28–60)/10# (5–30)	39.38	49 (3–265)

Study	Time from initial RT to ReRT (months)	Overall survival (months)	Progression-free survival (months)	Radiation necrosis	CTCAE grade 3–5 toxicities	Treatment-related deaths	Quality of life
van Linde [28]	NR	RT = 9.2 Syst = 7.3	RT = 7.7 Syst = 4.3	NR	NR	NR	NR
Ciammella [29]	10.8 (6–54)	RT = 9.5 Syst = 5.5	NR	NR	Total = 0%	NR	NR
Socha [30]	NR	RT = 4.3 Syst = 5.3	NR	NR	NR	NR	NR
Kim [31]	NR	RT = 9.2 Syst = 5.6 Comb = 15.5	RT = 3.6 Syst = 2.3 Comb = 6.0	NR	NR	NR	NR
Bovi [32]	NR	Syst = 9 Comb = 16	Syst = .4 Comb = 12	NR	NR	NR	NR
Bergman [33]	NR	Syst = 4.8 Comb = 7.2	Syst = 1.8 Comb = 5.1	Comb; 0%	Syst; 4/17 (23.5%) Comb; 6/18 (33.3%)	NR	NR
Yasuda [34]	18.7 (1.3–438)	Syst = 7.6 Comb = 10.4	Syst = 4.8 Comb = 5.6	Comb; 2/29 (6.9%), resolved after adjuvant Bev	Syst; 0/29 (0%) Comb; 3/29 (10.3%)	NR	NR
Schnell [35]	19.2	Syst = 6.6 Comb = 13.1	NR	0%	syst = 4/30 (13.3%), Comb (concurrent-maintenance) = 3/28 (10.7%) Comb (concurrent) = 1/47 (2.1%)	NR	NR
Lee [26]	NR	NR	Syst = 4.26 Comb = 5.53	NR	Total = 20/203 (9.9%)	Total = 0 (0%)	NR
Tsien [27]	Criteria; >/6 months	Syst = 9.7 Comb = 10.1	Syst = 3.8 Comb = 7.1	0%	Syst = 20/84 (23.8%) Comb = 22/86 (25.6%)	Syst (0%) Comb; 1/86 (1.2%)	NR
Yazici [36]	15 (5–45)	RT = 9.7 Comb = 16.8	NR	Total = 1/37 (2.7%)	NR	NR	NR
Wick [37]	Criteria; >/8 months	RT = 11.5 Comb = 11.5	RT = 2.5 Comb = 4.5	NR	NR	NR	No difference between the two groups over time in any scales (EORTC QLQ with EORTC QLQ-C15 PAL questionnaire and brain module EORTC QLQBN-20.)
Miwa [38]	NR	RT = 6 Comb = 12	RT = 5 Comb = 6	Total = 2/21 (9.5%)	Total = 1/21 (4.8%)	NR	NR
Lovo [39]	4.5 (1–44)	RT = 7 Comb = 12	NR	NR	NR	NR	NR
Baehr [40]	10 (3–54)	RT = 4 Comb = 12	RT = 2 Comb = 4.3	Total = 0%	ReRT; 2/10 (20%) Comb; 1/30 (3.33%)	NR	NR
Hasan [41]	NR	NR	NR	Total = 0%	NR	NR	NR
Conti [42]	Criteria; >/6 months	RT = 7 Comb = 12	RT = 4 Comb = 7	RT; 0/11 (0%) Comb = 1/12 (8.3%) Total = 4.30%	Comb; Grade 3 haematological toxicity in >/33%	reRT; 0/11 (0%) Comb; 1/12 (8.3%)	NR
Fogh [43]	NR	reRT = 10 Comb = 11	NR	NR	Total = 1/147 (0.7%)	NR	NR
Eberle [44]	10 (3–154)	reRT = 6 Comb = 14 months	NR	Total = 2/30 (6.7%)	Total = 8/30 (26.7%)	NR	NR
Saeed [45]	NR	NR	NR	NR	Total (acute); 1/45 (2.2%) Total (late); 4/45 (8.8%)	NR	NR
Scartoni [46]	21.3 (5–96) Criteria; >/3 months	NR	NR	Total = 3/33 (9.10%)	NR	NR	Concomitant chemotherapy significantly impacted the EORTC QLQC30_Physical values; patients who received RT and concomitant TMZ showed a more relevant decrease during analysed time points (p = 0.018)
Shen [47]	27.6 (4.8–214.2)	reRT = 11.5 mths, reRT + TMZ = 10.8 mths, RT + BEV = 6.6 mths, RT + TMZ + BEV = 4.8 mths	NR	Total = 4/118 (3.4%)	Total CNS Toxicity-11/118 (9.3%; all acute, no late toxicities) Comb-less than 10% of patients had grade 3 + anaemia (1%), leukopenia (5%), neutropenia (6%), and thrombocytopenia (8%), but 30% of patients experienced grade 3 + lymphopenia	NR	NR
Cheon, 2018 [48]	NR	reRT = 11.6 mths Comb =14.7 mths	reRT = 5.2 Comb = 4.5	NR	NR	NR	NR
Park, 2012 [49]	NR	reRT = 12.2 mths reRT + BEV = 17.9 mths	reRT = 6.7 reRT + BEV = 14.9	ReRT; NR ReRT + BEV; 1 (9%)	ReRT + BEV; Grade 3 toxicity-1/11 (9%)	NR	NR
Chan, 2020 [50]	NR	reRT = 4.4 mths reRT + Bev = 7.9 mths	NR	reRT; 4/6 (66.7%) reRT + Bev-0/61 (0%) Total = 6.0%	ReRT; 3/6 (50%)-RN (improved after receiving Bev)	NR	NR
Hundsberger [51]	40.9 (6.1–387.9)	reRT = 14.3 months reRT + BEV = 8.4	reRT = 3.7 reRT + BEV = 5.7	ReRT; 1/4 (25%) ReRT + BEV-0/10 (0%) Total = 7.10%	Total = 0%	reRT = 0/4 (0%) reRT + BEV = 1/10 (10%)	NR
Guan [52]	NR	1-year OS rates in grade 4 patients; reRT; 56% reRT + Bev; 77.3%	NR	NR	Total = 0%	NR	NR
Fleischmann [18]	reRT = 18 (5–182) reRT + Bev = 17 (4–265)	reRT = 9 mths reRT + BEV = 9 mths	reRT = 5 reRT + BEV = 5	ReRT = 5/37 (13.5%) ReRT + BEV = 6/124 (4.8%) Total = 6.80%	BEV-related = 12/124 (9.7%)	NR	NR
Cuneo [53]	NR	reRT = 3.9 mths reRT + BEV = 11.2 mths	reRT = 2.1 reRT + Bev = 5.2	Reported in all HGG patients (21 (no Bev), 42 (Bev)); Re-RT; 4/21 (19%) ReRT + BEV; 2/42 (4.7%) Total = 9.50%	Reported in all HGG patients (21 (no Bev), 42 (Bev)); ReRT; 4/21 (19%) ReRT + BEV; 4/42 (10%)	NR	NR
Helis [25]	41.3	NR	NR	NR	NR	NR	NR
Youland [54]	NR	NR	NR	ReRT = 4 patients (19%) ReRT + BEV = 0 (0%) Total = 8.30%	Total = 3/48 (6%)	NR	NR

*Syst* systemic therapy, *ReRT* reirradiation, *Comb* combination therapy, *Bev* bevacizumab, *TMZ* temozolomide, *Chemo* chemotherapy, *HF-SRT* hypofractionated stereotactic radiotherapy, *RT* radiotherapy, *SRS* stereotactic radiosurgery, *Gy* gray, *#* fractions, *cc* cubic centimetres, *RN* radiation necrosis, *HGG* high-grade glioma, *NR* not reported

### Risk of bias

**Fig. 2:**
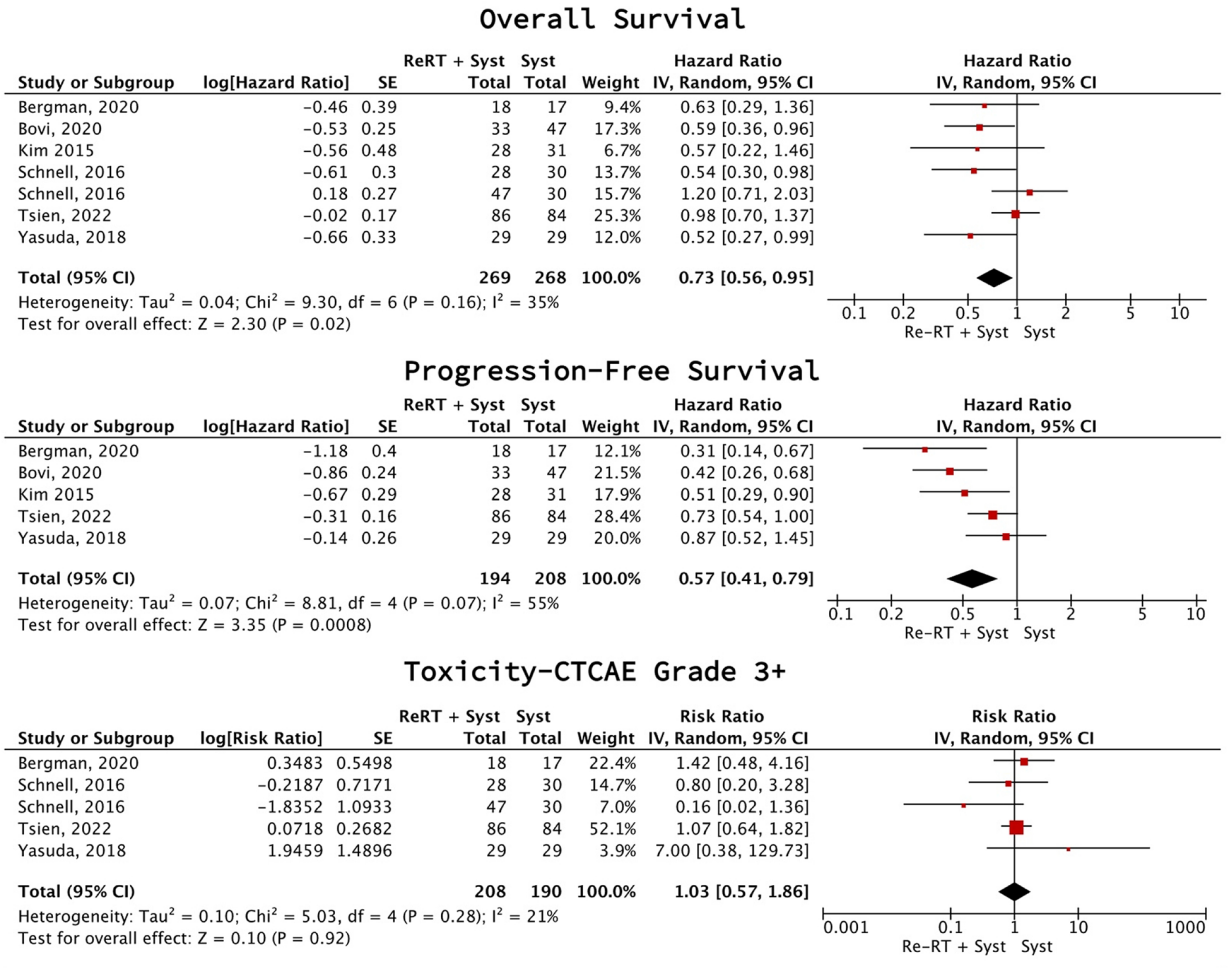
Combination therapy vs systemic therapy meta-analysis (rHGG)

**Fig. 3:**
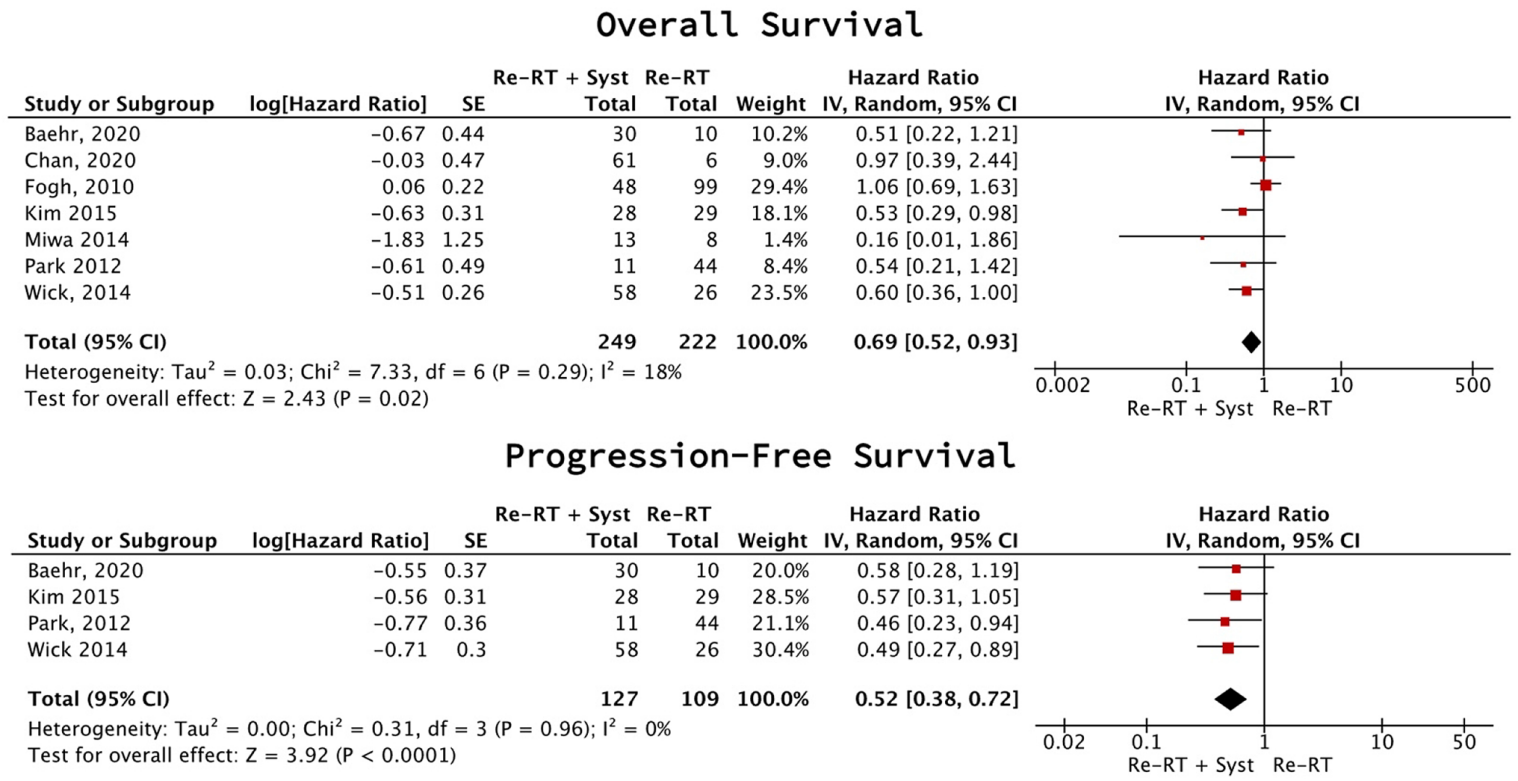
Combination therapy vs reirradiation meta-analysis (rHGG)

**Fig. 4:**
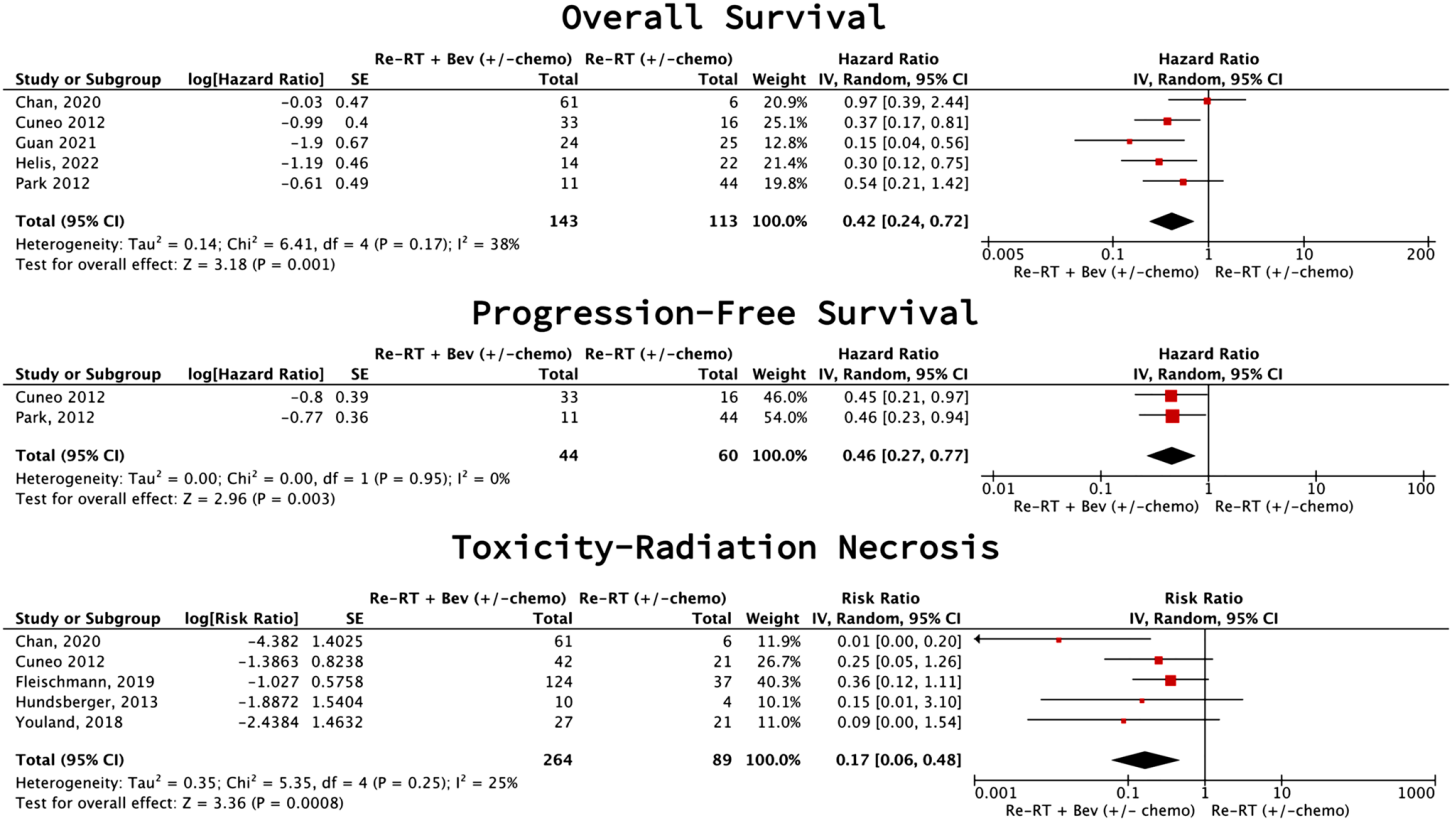
Bevacizumab-based combination therapy vs reirradiation with/without non-bevacizumab-based systemic therapy meta-analysis (rHGG)

The RoB assessment is demonstrated in the Supplementary Material; Figs. 2, 3, 4, 5. The 3 RCTs [27, 33, 37] were of low RoB. Of the 28 non-randomised studies, 19 were of moderate RoB [18, 25, 28, 29, 31, 32, 34, 35, 38, 40, 43, 45, 48–54], and 9 were of serious RoB (and hence excluded from meta-analyses) [26, 30, 36, 39, 41, 42, 44, 46, 47].

### Treatment outcomes & meta-analyses: rHGG

#### Reirradiation vs systemic therapy

In the reRT group, the median PFS ranged from 3.6 to 7.7 months, while the median OS ranged from 4.3 to 9.5 months. In the systemic therapy group, the median PFS ranged from 2.3 to 4.3 months, while the median OS ranged from 5.3 to 7.3 months. No grade 3–5 toxicities were reported in this group (Table 2).

There was no difference in PFS (2 studies [28, 31], 185 participants; HR 0.87 (95% CI 0.61–1.22), p = 0.41, I2 = 0%; very low certainty) and OS (3 studies [28, 29, 31], 237 participants; HR 0.94 (95%CI 0.67–1.31), p = 0.70, I2 = 0%; very low certainty) (Fig. 1). A meta-analysis could not be conducted for AEs due to insufficient reporting.

#### Combination therapy vs systemic therapy

In the combination therapy group, the median PFS ranged from 5.1 to 12 months, while the median OS ranged from 7.2 to 16 months. In the systemic therapy group, the median PFS ranged from 1.8 to 4.8 months, while the median OS ranged from 4.8 to 9.7 months. In the combination therapy group rates of grade 3 + AEs ranged from 2.1 to 33.3% while in the systemic therapy group rates ranged from 0 to 23.8%. 1 treatment-related death was reported with combination therapy (Table 2).

Combination therapy improved PFS (5 studies [27, 31–34], 402 participants; HR 0.57 (95% CI 0.41–0.79), p = 0.0008, I2 = 55%; low certainty) and OS (6 studies [27, 31–35], 537 participants; HR 0.73 (95% CI 0.56–0.95), p = 0.02, I2 = 35%; low certainty), and there was no difference in grade 3 + toxicities (4 studies [27, 33–35], 398 participants; RR 1.03 (95% CI 0.57–1.86), p = 0.92, I2 = 21%; very low certainty) (Fig. 2).

Subset analyses of studies comparing combination therapy with bevacizumab-based systemic therapy to bevacizumab-based systemic therapy alone are demonstrated in Supplementary Material; Fig. 6. Subset analyses of only RCTs are demonstrated in Supplementary Material; Fig. 7.

#### Combination therapy vs reirradiation

In the combination therapy group, the median PFS ranged from 4.3 to 14.9 months, while the median OS ranged from 4.8 to 17.9 months. In the reRT group, the median PFS ranged from 2 to 6.7 months, while the median OS ranged from 4 to 14.3 months. In the combination therapy group, rates of grade 3 + AEs ranged from 0 to 33%, while in the reRT group rates ranged from 0 to 50.0%. RN rates ranged from 0 to 9.5%. Two treatment-related deaths were reported in the combination therapy group (Table 2).

Combination therapy improved PFS (4 studies [31, 37, 40, 49], 236 participants; HR 0.52 (95% CI 0.38–0.72), p < 0.0001, I2 = 0%; low certainty) and OS (7 studies [31, 37, 38, 40, 43, 49, 50], 471 participants; HR 0.69 (95% CI 0.52–0.93), p = 0.02, I2 = 18%; low certainty) (Fig. 3).

#### Bevacizumab-based combination therapy vs reirradiation with/without non-bevacizumab-based systemic therapy

In the bevacizumab-based combination therapy group, the median PFS ranged from 5 to 14.9 months, while the median OS ranged from 7.9 to 17.9 months. In the reRT without bevacizumab group, the median PFS ranged from 2.1 to 6.7 months, while the median OS ranged from 3.9 to 14.3 months. RN rates in the bevacizumab-based combination therapy group ranged from 0 to 9%, while the RN rates in the reRT without bevacizumab group ranged from 13.5 to 66.7%. Rates of grade 3 + AEs ranged from 0 to 10% in the bevacizumab-based combination therapy group while they ranged from 0 to 50% in the reRT without bevacizumab group. 1 treatment-related death was reported in the bevacizumab-based combination therapy group (Table 2).

Combining reRT with bevacizumab-based systemic therapy improved PFS (2 studies [49, 53], 104 participants; HR 0.46 (95% CI 0.27–0.77), p = 0.003, I2 = 0%; low certainty) and OS (5 studies [25, 49, 50, 52, 53], 256 participants; HR 0.42 (95% CI 0.24–0.72), p = 0.001, I2 = 38%; low certainty), while reducing RN (5 studies [18, 50, 51, 53, 54], 353 participants; RR 0.17 (95% CI 0.06–0.48), p = 0.0008, I2 = 25%; low certainty) (Fig. 4).

### Subset analysis: rGBM

In the reRT vs systemic therapy group, there was no difference in PFS (2 studies [28, 31], 185 participants; HR 0.87 (95% CI 0.61–1.22), p = 0.41, I2 = 0%) and OS (3 studies [28, 29, 31], 237 participants; HR 0.94 (95% CI 0.67–1.31), p = 0.70, I2 = 0%) (Supplementary Material; Fig. 8). In the combination therapy vs systemic therapy group, combination therapy improved PFS (2 studies [27, 31], 229 participants; HR = 0.66 (95% CI 0.49–0.91), p = 0.01, I2 = 15%), although there was no difference in OS (2 studies [27, 31], 229 participants; HR 0.90 (95% CI 0.62–1.32), p = 0.60, I2 = 11%) (Supplementary Material; Fig. 9). In the combination therapy vs reRT group, combination therapy improved PFS (4 studies [31, 37, 40, 49], 229 participants; HR 0.52 (95% CI 0.38–0.72), p < 0.0001, I2 = 0%) and OS (5 studies [31, 37, 38, 40, 49], 257 participants; HR 0.55 (95% CI 0.39–0.76), p < 0.0003, I2 = 0%) (Supplementary Material; Fig. 10). Combining reRT with bevacizumab-based systemic therapy improved PFS (2 studies [49, 53], 104 participants; HR 0.46 (95% CI 0.27–0.77), p = 0.003, I2 = 0%) and OS (4 studies [25, 49, 52, 53], 189 participants; HR 0.34 (95% CI 0.21–0.55), p < 0.00001, I2 = 0%) (Supplementary Material; Fig. 11).

### Publication bias

Funnel plots are presented in the Supplementary Material; Figs. 12, 13, 14, 15. On visual inspection, there is low evidence of bias in most funnel plots. There is some concern for publication bias for toxicities (RN) in the bevacizumab-based combination therapy vs reirradiation with/without non-bevacizumab-based systemic therapy group (Supplementary Material; Fig. 15).

### GRADE approach

The certainty of evidence assessment is summarised in Supplementary Material; Table 1.

## Discussion

Compared to reRT alone, combination therapy improved OS and PFS. While there was insufficient information to conduct a meta-analysis comparing AEs, Kazmi et al. reported no significant differences in toxicity between reRT alone and combination therapy (5% vs 9% respectively, p = 0.22) [9]. Further RCTs are required to confirm the survival benefit and safety of combination therapy compared to reRT alone.

Particularly, the addition of bevacizumab to reRT with/without non-bevacizumab-based systemic therapy improved OS and PFS and reduced RN. Grade 3 + AEs were also lower with bevacizumab compared to without (0–10% vs 0–50%, respectively), largely secondary to decreased rates of Grade 3 + RN. These findings differ from the AVAglio [55] and RTOG 0825 [56] trials which explored the supplementation of the Stupp protocol [2] with bevacizumab in primary GBM. Both RCTs found bevacizumab resulted in a PFS improvement and a modest increase in grade 3 + AEs. However, as neither trial demonstrated an OS benefit, bevacizumab is not routinely used for primary GBM. Bevacizumab is, however, commonly utilised for recurrent GBM in the absence of proven OS benefits due to reported PFS improvements and steroid sparing effects, both which are postulated to improve QoL [14]. This review supports further investigation into the addition of bevacizumab to reRT in the recurrent HGG context given the demonstrated OS and PFS improvements and lower rates of grade 3 + AEs secondary to decreased rates of grade 3 + RN.

The addition of bevacizumab to reRT may also allow for safe dose escalation and for the treatment of larger volume disease due to its radioprotective properties. While bevacizumab is routinely used for the treatment of RN [57], it is not regularly used as a prophylactic agent [18]. In 2012, Sminia and Mayer found that RN occurred with a cumulative EQD2 dose > 100 Gy for conventional RT, > 105 Gy for HFSRT, and > 135 Gy for SRS. [58] In this review, 17 studies reported RN rates ranging from 0 to 9.5% (Supplementary Material; Table 2). 9 of these 17 studies escalated their cumulative EQD2 (a/b = 2) beyond Sminia and Mayer’s recommendation. Importantly, of these 9 studies, the rate of RN in the subset of patients that received bevacizumab with reRT was 0%, while the rate of RN in the subset of patients that did not receive bevacizumab ranged from 4.3 to 25.0%. Hence, bevacizumab may allow for safe dose escalation with acceptable rates of RN. Further studies are required to confirm if dose escalation confers improved local control or survival outcomes. RN is also a concern in the treatment of large volume disease. In 2021, Minniti et al. recommended SRS or high dose HFSRT (≥ 5 Gy/#) for smaller volume tumours (≤ 15 cc), high-dose HFSRT (≥ 5 Gy/#) for 8.5–34 cc tumours, and conventional RT or moderately HFSRT (1.8–3.5 Gy/#) for larger tumours (33–145 cc) to appreciate a low risk of RN [7]. In this review, 10 of 17 studies reporting RN rates also reported median PTV (Supplementary Material; Table 2). Of these 10 studies, 8 had median PTV > 34 cc. Importantly, in these 8 studies, the rate of RN in the subset of patients that received bevacizumab with reRT ranged from 0 to 4.8%, while the rate of RN in the subset of patients that did not receive bevacizumab ranged from 0 to 66.7%. Notably, all 8 studies utilised conventional RT or moderately HF-SRT, as recommended by Minniti et al. for larger volume tumours. Hence, reRT with concomitant bevacizumab may allow for the safer treatment of larger volume disease with acceptable rates of RN, particularly if the appropriate fractionation schedule is utilised.

Compared to systemic therapy alone, combination therapy (particularly with bevacizumab-based systemic therapy) improved OS and PFS with no difference in grade 3 + AEs. Two RCTs investigated bevacizumab with/without reRT in rHGG [27, 33]. Tsien et al. [27] compared bevacizumab with/without HFSRT (35 Gy/10#) in patients with bevacizumab-naïve rGBM. The study found that HFSRT improved PFS and 6-month PFS rates, though no improvements in OS were observed. However, due to low accrual, the study was amended to extend eligibility resulting in the inclusion of a large number of patients less likely to experience a survival benefit from focal HFSRT. Bergman et al. [33] compared bevacizumab-based systemic therapy with/without intervening HFSRT (32 Gy/4#) in patients with bevacizumab-resistant rHGG. Patients assigned to intervening HFSRT reported improved PFS and a nonsignificant improvement in OS, despite the study also failing to meet accrual goals to detect an OS difference. Interestingly, while Bergman et al. targeted FLAIR abnormalities in their CTV delineation, Tsien et al. did not. FLAIR abnormalities are non-enhancing regions that likely contain microscopic disease. Studies targeting FLAIR abnormalities have demonstrated improved locoregional control, suggesting that the deterioration of patients with rGBM may be due to insufficient reRT dose to these regions [59]. Further RCTs comparing systemic therapy with combination therapy, particularly with bevacizumab-based systemic therapy, are required. Importantly, the limitations of previous RCTs must be addressed; namely the inadequate accrual of appropriate patients, and the exclusion of FLAIR abnormalities from CTV delineation. Studies comparing QoL and neurocognitive function are needed as well.

Subset analyses of rGBM-only studies demonstrated similar improvements in OS and PFS with combination therapy across all comparative treatment groups, though no significant OS benefit was observed compared to systemic therapy alone. These findings are of particular clinical significance as there is no widely accepted standard-of-care for this patient cohort with the poorest prognosis [17]. Hence, further RCTs comparing combination therapy to systemic therapy or reirradiation alone in patients with rGBM are especially warranted.

This review has some limitations. There were insufficient studies to conduct a meta-analysis on QoL, and the impact of resection on survival could not be ascertained. Studies were also mainly of retrospective cohort methodology, hence conferring a greater risk of confounding and selection bias potentially favouring combination treatment. Furthermore, most studies incompletely reported molecular information (IDH/MGMT) and were conducted prior to the changes in WHO glioma classification in 2021. Of note, grading largely informs the management approach at initial diagnosis and relapse, and MGMT methylation status is a vital prognosticator in an era where most gliomas are treated with alkylating agents [4]. Further RCTs are therefore required to address these limitations and confirm this review’s findings. Additionally, the diagnosis of tumour progression/recurrence varied between studies; radiological diagnosis vs biopsy-proven. Notably, it is difficult to differentiate tumour progression with treatment-related changes using conventional MRI [60].

## Conclusion

This review found that combination therapy may improve OS and PFS with acceptable toxicity in select patients with rHGG compared to reirradiation or systemic therapy alone. Hence, further RCTs are warranted although the limitations of previous RCTs must be addressed; namely the inadequate accrual of appropriate patients to detect an OS difference, and the exclusion of FLAIR abnormalities from CTV delineation. Additional studies comparing QoL and neurocognitive function are needed as well. This review also found that the addition of bevacizumab to reRT reduced RN and may allow for safer dose escalation and treatment of larger volume disease. Further studies are required to determine if dose escalation confers improved local control or survival outcomes.

### Supplementary Information

Below is the link to the electronic supplementary material.

Supplementary file1 (DOCX 3318 KB)

## Data Availability

Data will be made available by the corresponding author upon reasonable request.
