# Supplementary material for: Reirradiation versus systemic therapy versus combination therapy for recurrent high-grade glioma: a systematic review and meta-analysis of survival and toxicity
Source: J Neurooncol. 2023 Sep 21;164(3):505–24. doi: 10.1007/s11060-023-04441-0 (PMC10589175; doi:10.1007/s11060-023-04441-0)
Supplement: Supplementary file 1 — Supplementary file1 (DOCX 3318 KB) [file 11060_2023_4441_MOESM1_ESM.docx]

**Supplementary Material**


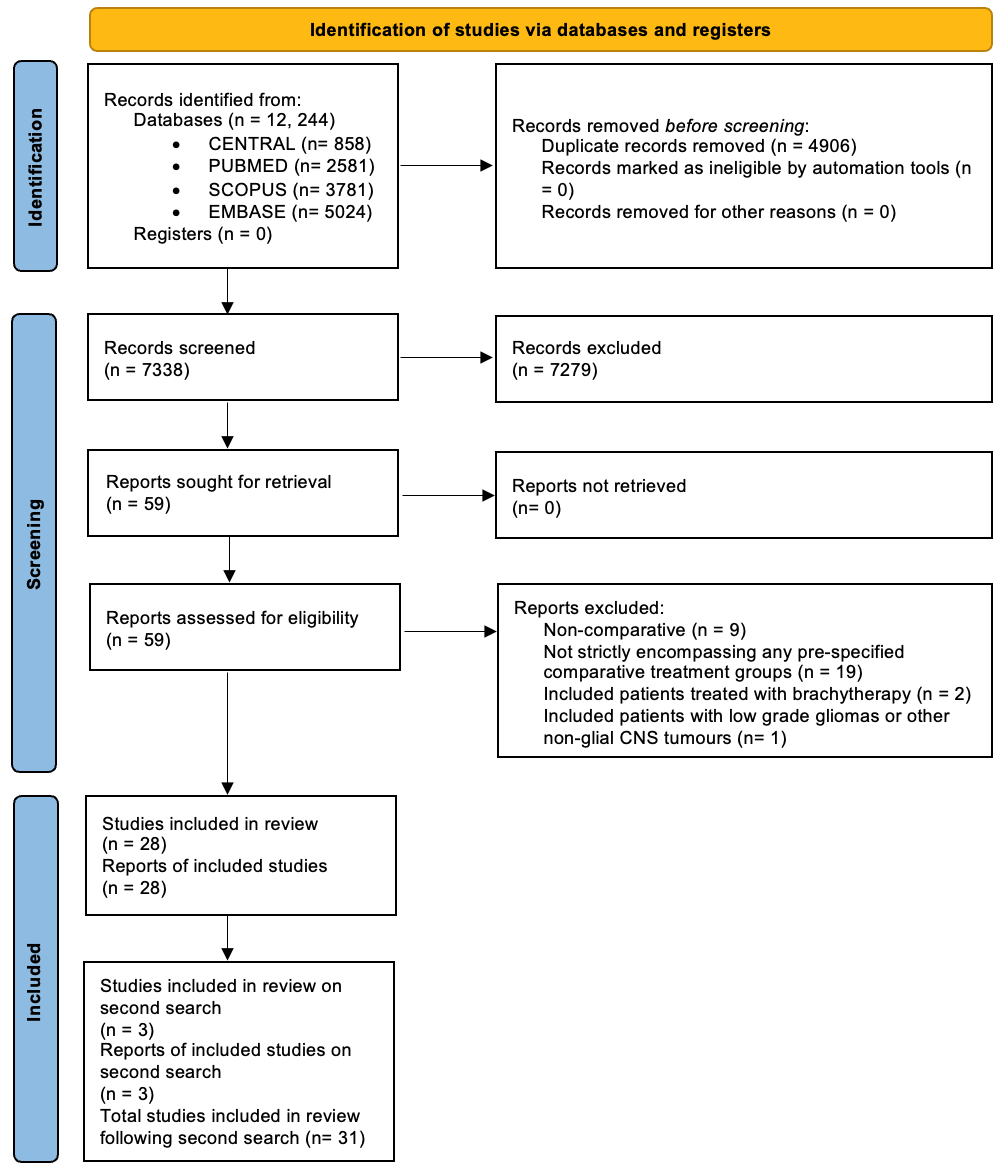


Figure 1: PRISMA Flow Diagram

**Risk of Bias Assessment**


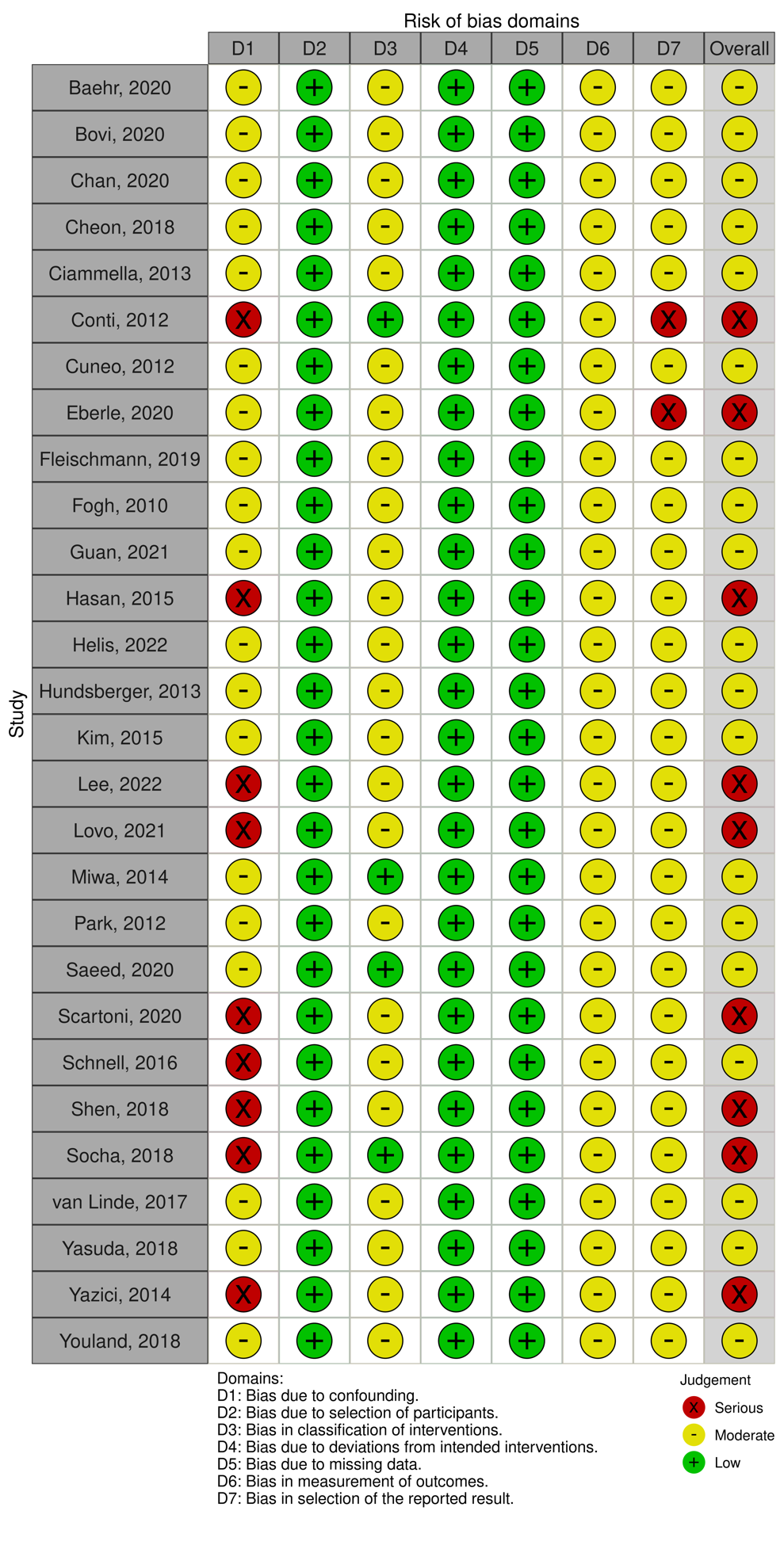


Figure 2: Risk of Bias Assessment (by study) in Non-Randomised Studies using ROBINS-I Tool


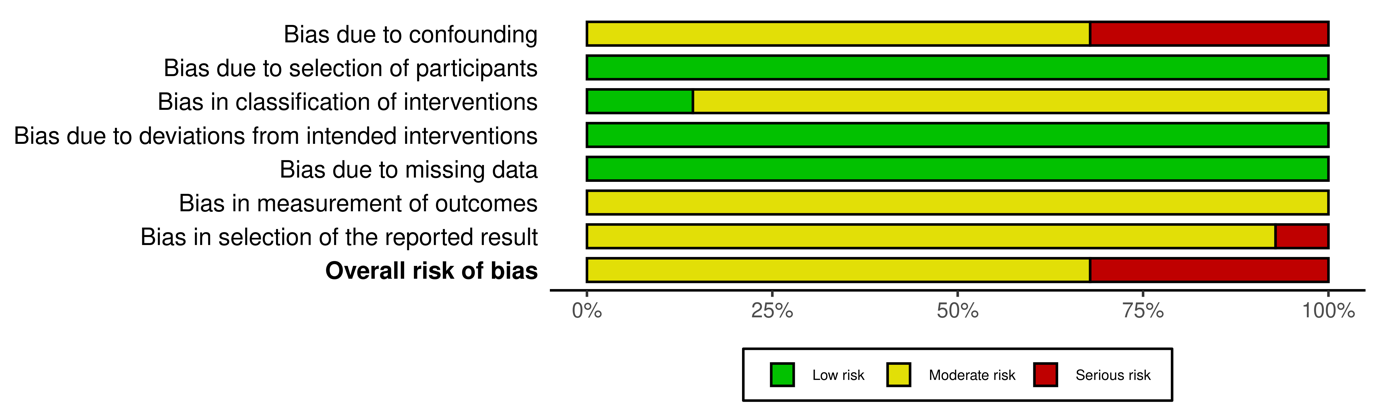


Figure 3: Risk of Bias Assessment (by domain) in Non-Randomised Studies using ROBINS-I Tool


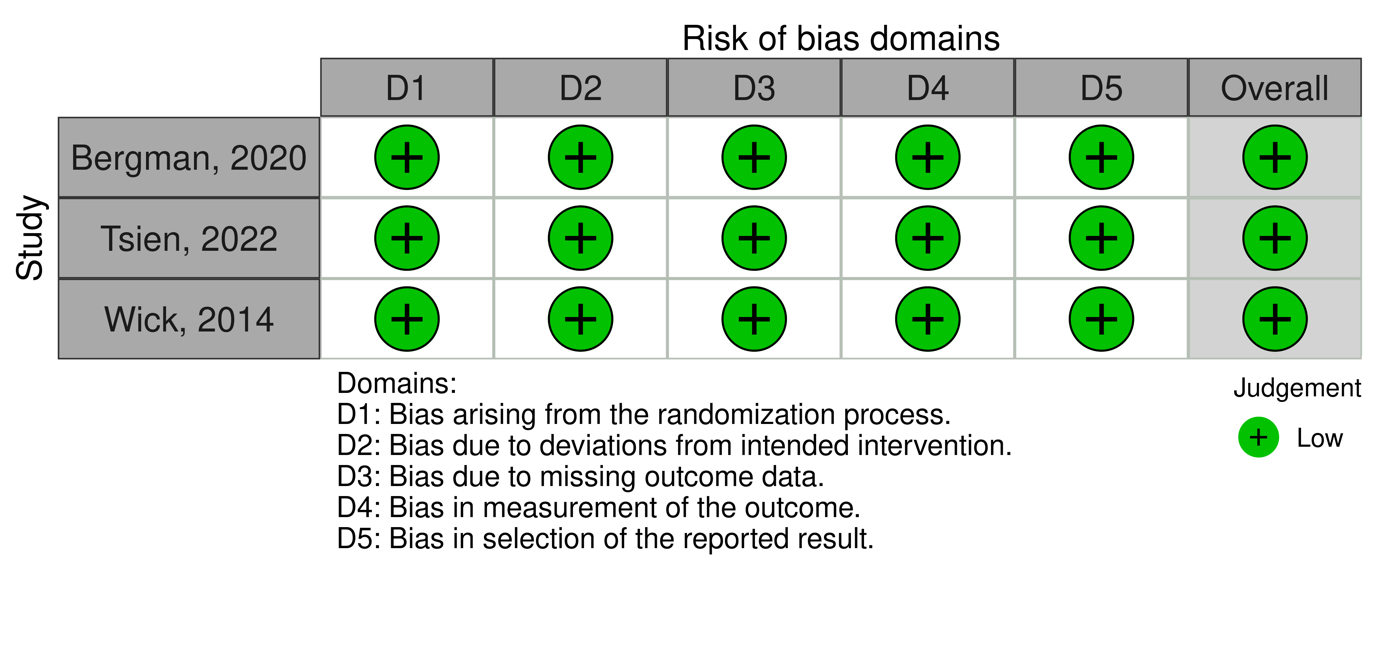


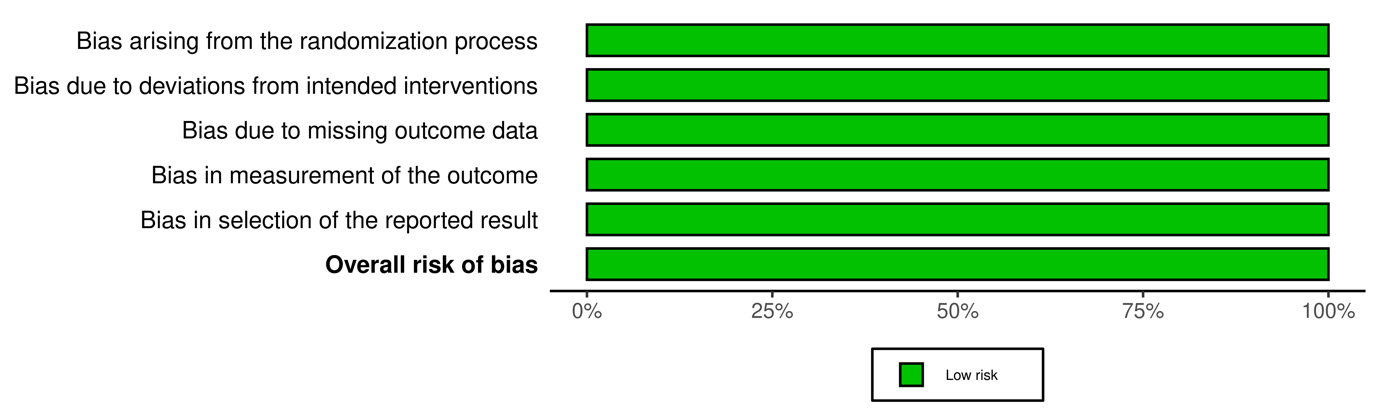
Figure 4: Risk of Bias Assessment (by study) in Randomised Studies using Cochrane RoB 2 Tool

Figure 5: Risk of Bias Assessment (by domain) in Randomised Studies using Cochrane RoB 2 Tool

**Chemotherapy-Specific (Sub-Analysis)**


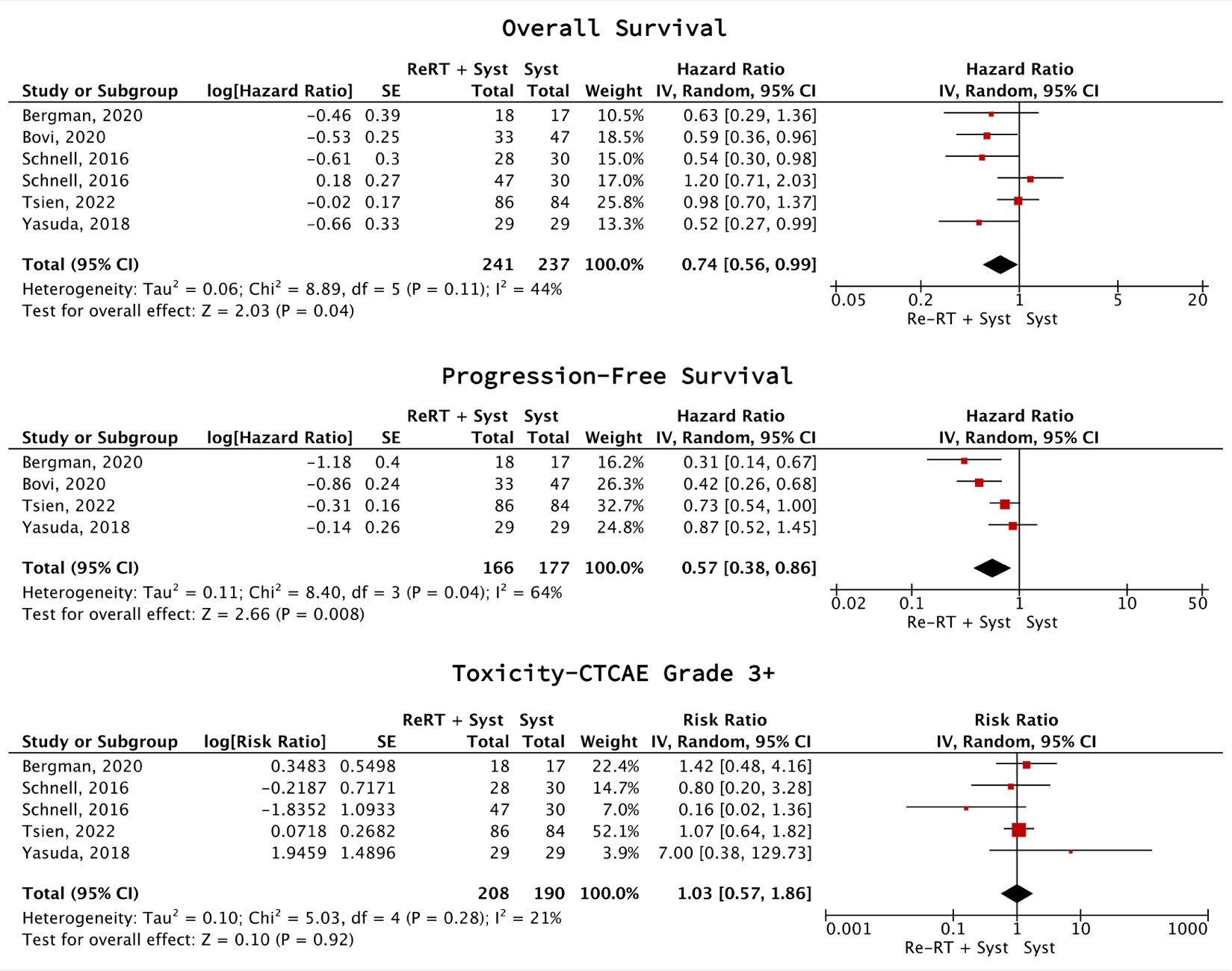


Figure 6: Bevacizumab-Based Combination Therapy vs Systemic Therapy Meta-Analysis

**RCT Only (Sub-Analysis)**


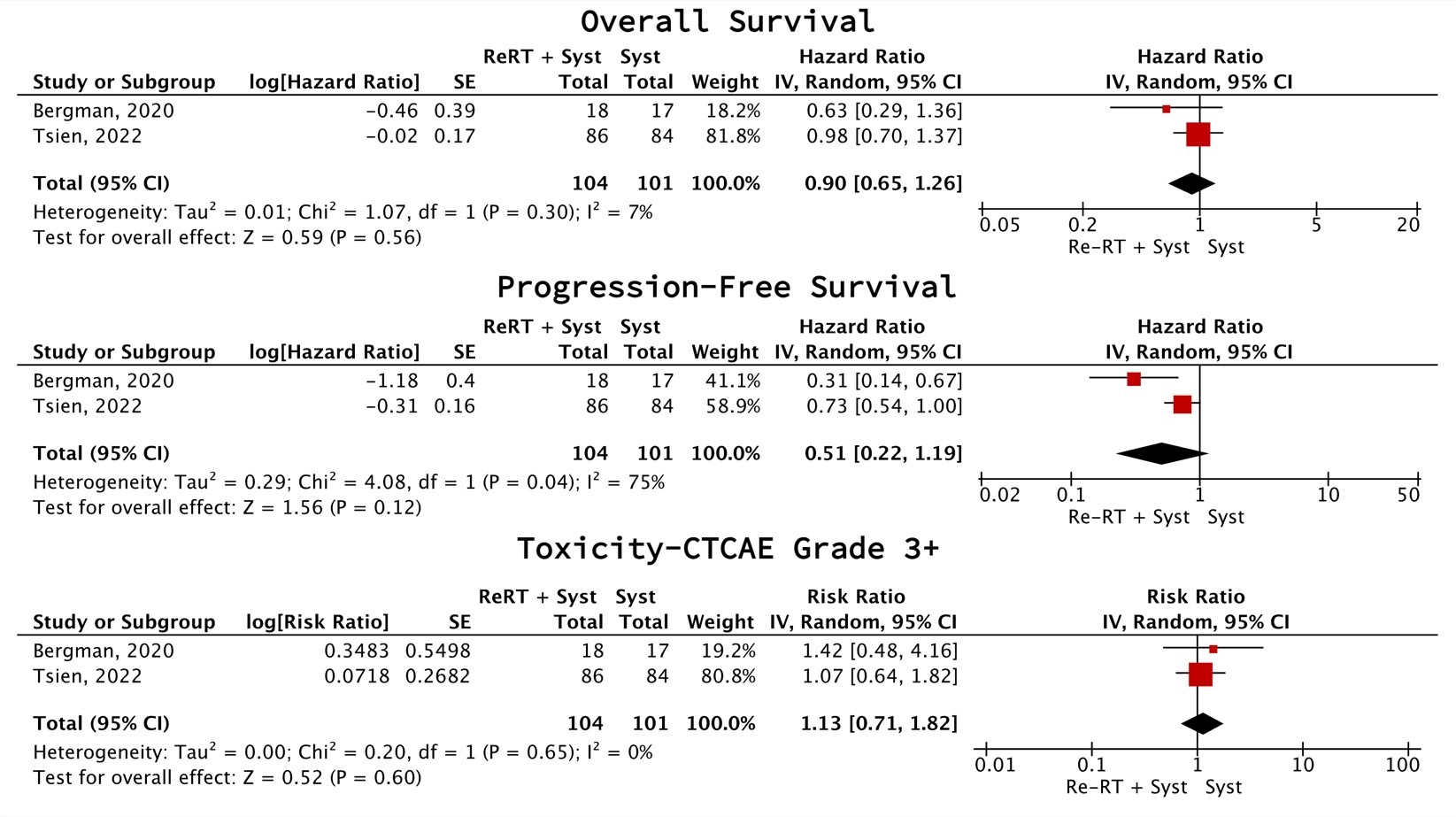


Figure 7: Combination Therapy vs Systemic Therapy Meta-Analysis (RCTs Only)

*
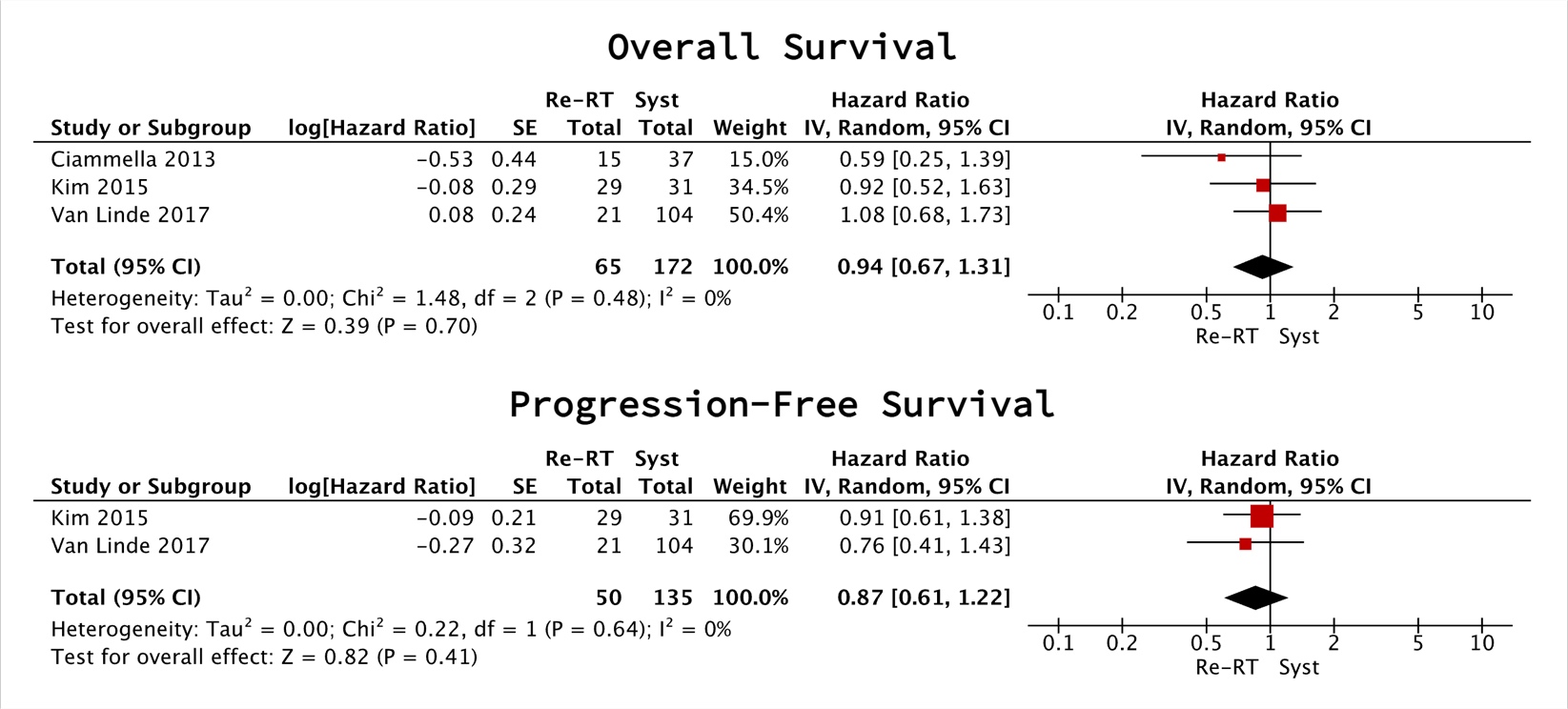
***GBM-Specific (Sub-Analysis)**

Figure 8: Reirradiation vs Systemic Therapy Meta-Analysis (rGBM)


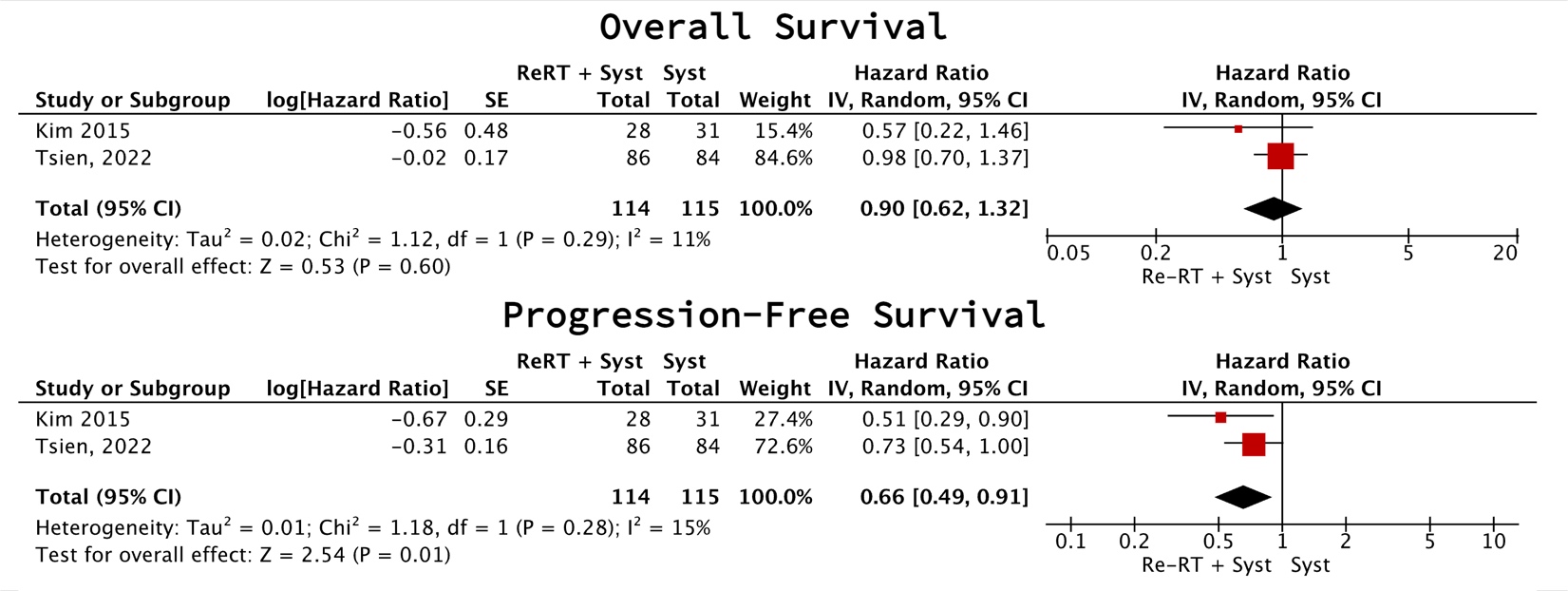


Figure 9: Combination Therapy vs Systemic Therapy Meta-Analysis (rGBM)

**
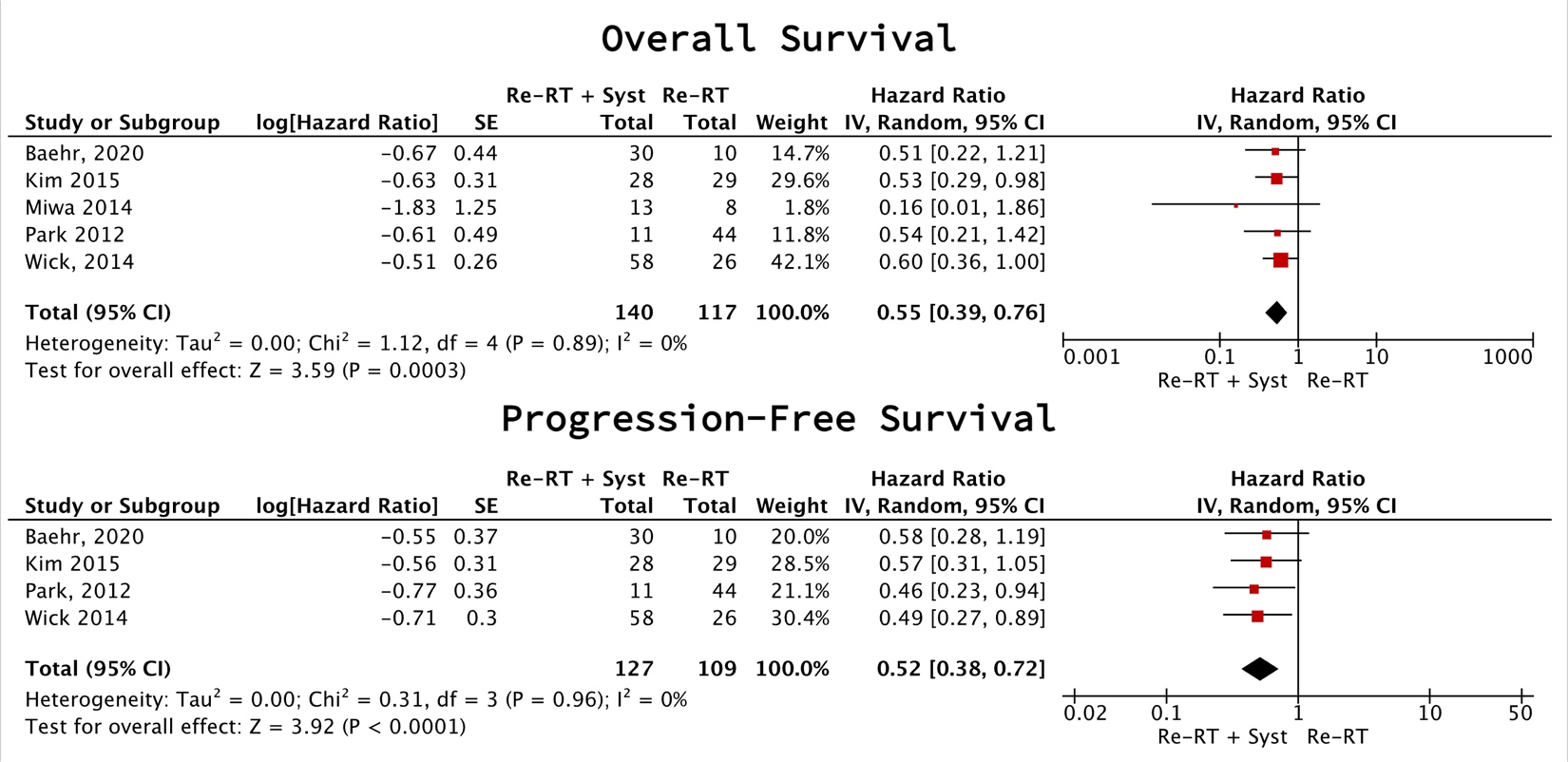
**Figure 10: Combination Therapy vs Reirradiation Meta-Analysis (rGBM)

*
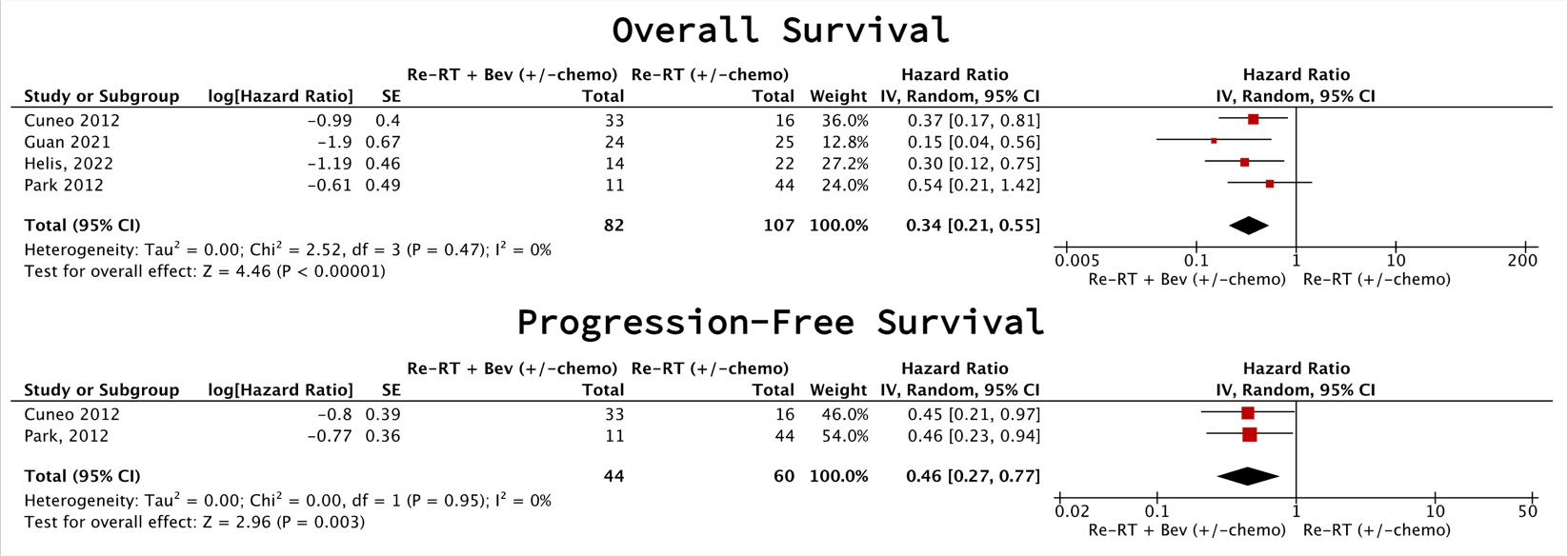
*

Figure 11: Bevacizumab-Based Combination Therapy vs Reirradiation with/without Non-Bevacizumab-Based Systemic Therapy Meta-Analysis (rGBM)

**Publication Bias**


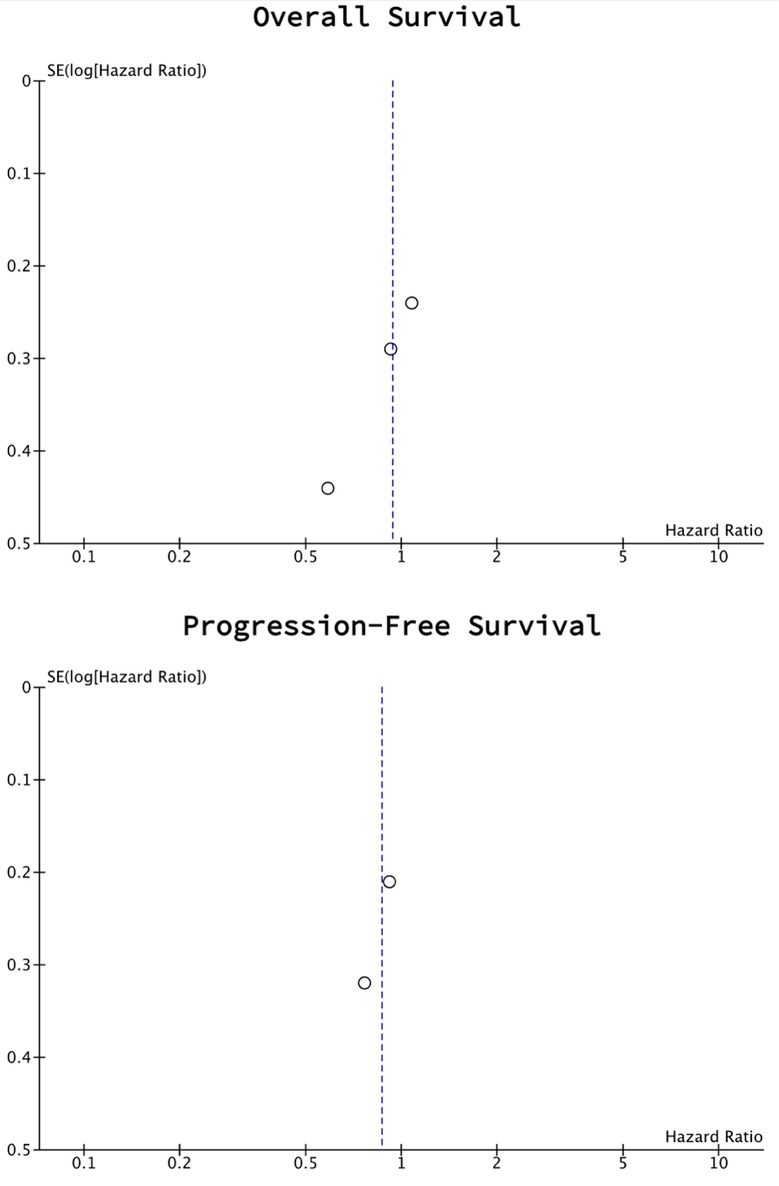


Figure 12: Reirradiation vs Systemic Therapy Funnel Plots (rHGG)


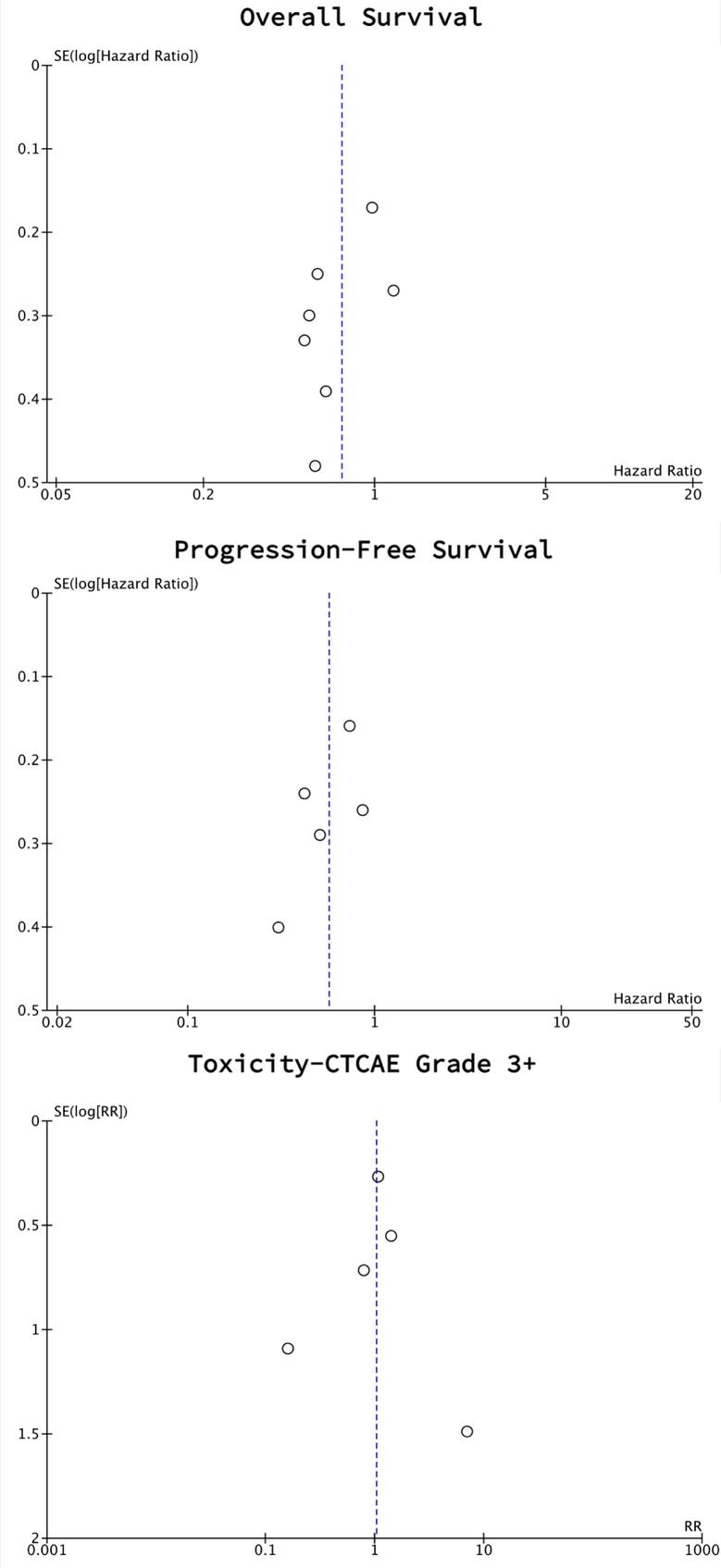


Figure 13: Combination Therapy vs Systemic Therapy Funnel Plots (rHGG)


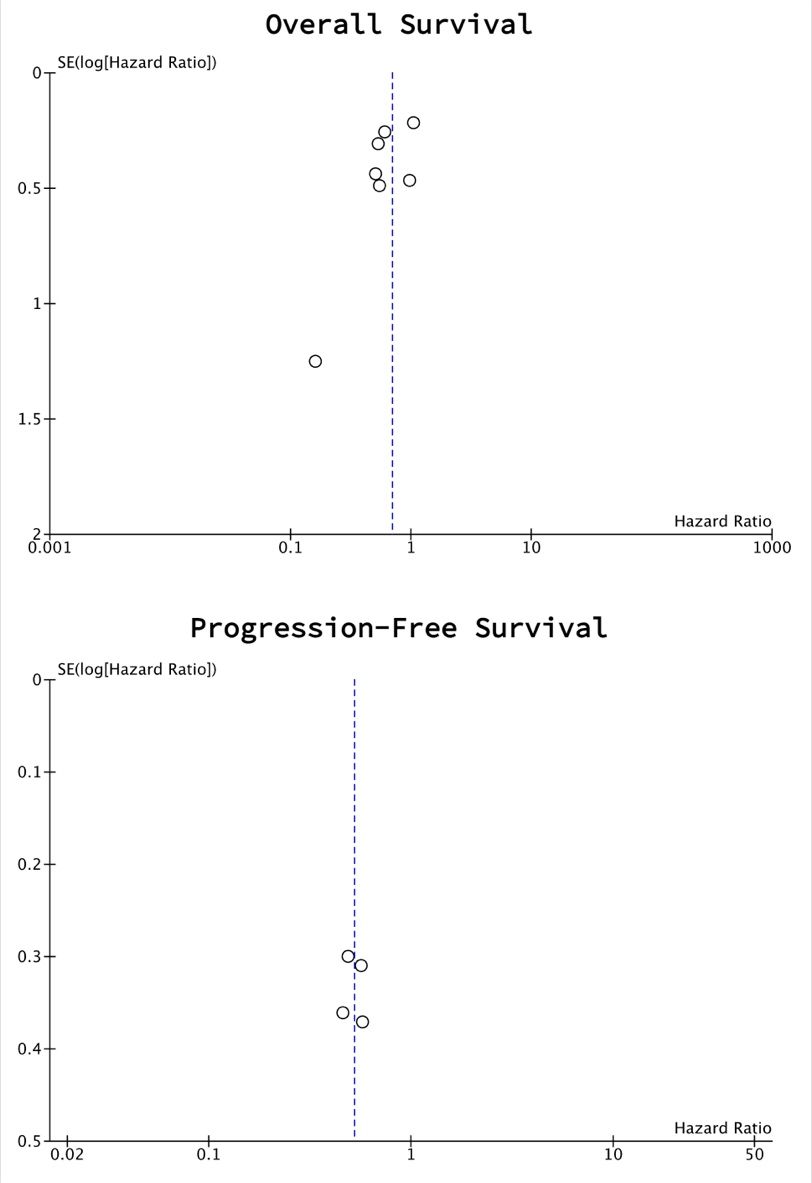


Figure 14: Combination Therapy vs Reirradiation Funnel Plots (rHGG)


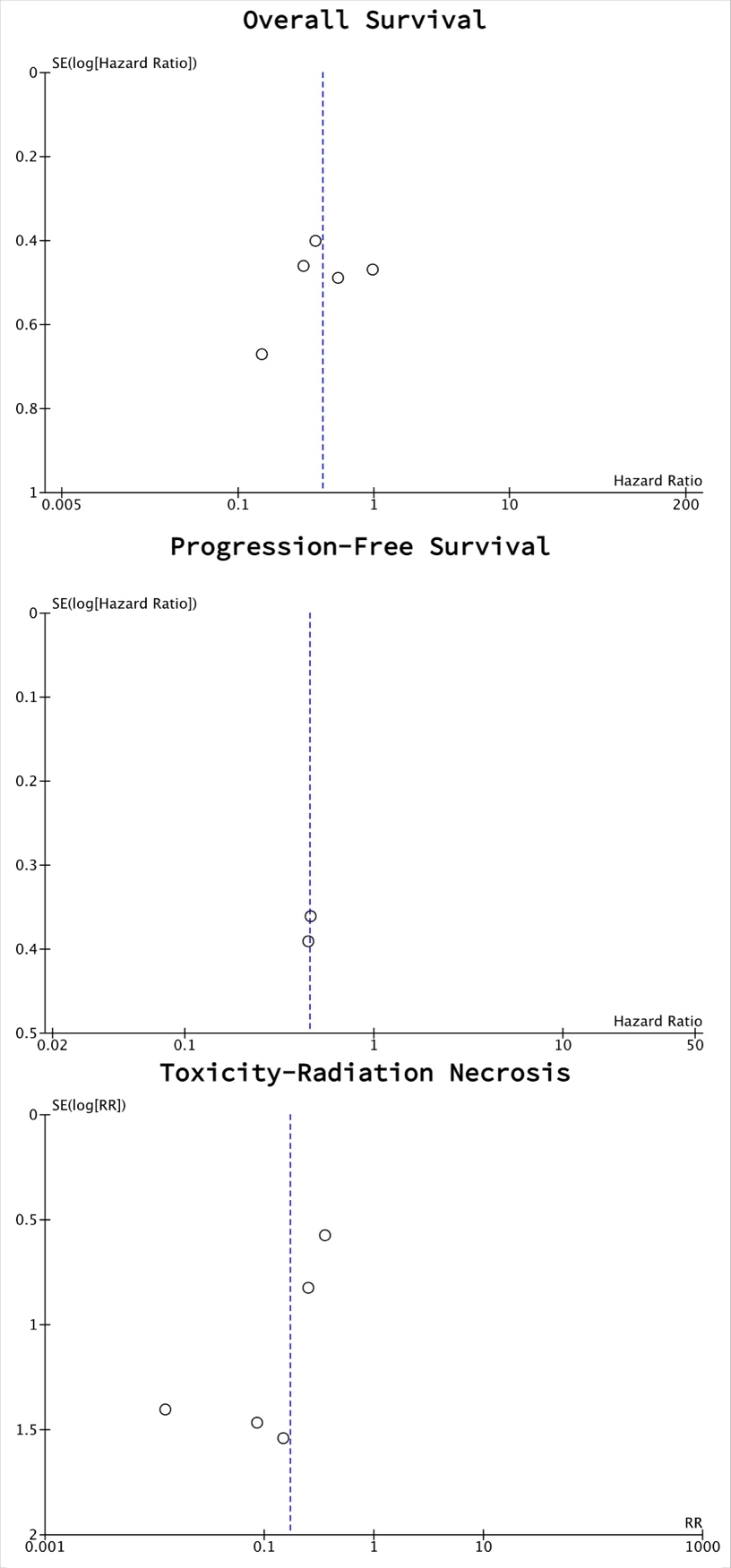


Figure 15: Bevacizumab-Based Combination Therapy vs Reirradiation with/without Non-Bevacizumab-Based Systemic Therapy Funnel Plots (rHGG)

Table 1: The GRADE Approach

| **Comparative Group** | **Outcomes of Interest** | **Studies Reporting Outcome** | **Study Participants** | **Relative Effect** | **Risk of Bias** | **Inconsistency** | **Indirectness** | **Imprecision** | **Publication Bias** | **Overall Certainty** |
| --- | --- | --- | --- | --- | --- | --- | --- | --- | --- | --- |
| ReRT vs Syst | PFS | 2 | 185 | HR 0.87 (95%CI 0.61-1.22) | Moderate | Not Serious | Not Serious | Serious (↓) ^a^ | Not Serious | Very Low |
|  | OS | 3 | 237 | HR 0.94 (95%CI 0.67-1.31) | Moderate | Not Serious | Not Serious | Serious (↓) ^b^ | Not Serious | Very Low |
| Comb vs Syst | PFS | 5 | 402 | HR 0.57 (95%CI 0.41-0.79) | Moderate | Not Serious | Not Serious | Not Serious | Not Serious | Low |
|  | OS | 6 | 537 | HR 0.73 (95%CI 0.56-0.95) | Moderate | Not Serious | Not Serious | Not Serious | Not Serious | Low |
|  | Grade 3+ Toxicities | 4 | 398 | RR 1.03 (95%CI 0.57-1.86) | Moderate | Not Serious | Not Serious | Serious (↓) ^c^ | Not Serious | Very Low |
| Comb vs ReRT | PFS | 4 | 236 | HR 0.52 (95%CI 0.38-0.72) | Moderate | Not Serious | Not Serious | Not Serious | Not Serious | Low |
|  | OS | 7 | 471 | HR 0.69 (95%CI 0.52-0.93) | Moderate | Not Serious | Not Serious | Not Serious | Not Serious | Low |
| Bev-Based Comb vs ReRT +/- Non-Bev-Based Syst | PFS | 2 | 104 | HR 0.46^d^ (95%CI 0.27-0.77) | Moderate | Not Serious | Not Serious | Not Serious | Not Serious | Low |
|  | OS | 5 | 256 | HR 0.42^e^ (95%CI 0.24-0.72) | Moderate | Not Serious | Not Serious | Not Serious | Not Serious | Low |
|  | Radiation Necrosis | 5 | 353 | HR 0.17^f^ (95%CI 0.06-0.48) | Moderate | Not Serious | Not Serious | Not Serious | Not Serious | Low |

Table 1: Certainty of Evidence; The GRADE Approach

^a^ Rated down due to wide confidence interval crossing 1.0

^b^ Rated down due to wide confidence interval crossing 1.0

^c^ Rated down due to wide confidence interval crossing 1.0

^d^ Did not rate up for large effect measure due to moderate RoB

^e^ Did not rate up for large effect measure due to moderate RoB

^f^ Did not rate up for very large effect measure due to moderate RoB & some concern of publication bias

Abbreviations: PFS, Progression-Free Survival; OS, Overall Survival; HR, Hazard Ratio; RR, Risk Ratio; CI, Confidence Interval

Table 2: Radiation Necrosis

| **Study** | **Reirradiation Group** | **Patients Receiving Bevacizumab** | **Planning Target Volume (cc)** | **Time to Reirradiation (months)** | **Cumulative EQD2 (a/b=2) (Gy)** | **Radiation Necrosis** |
| --- | --- | --- | --- | --- | --- | --- |
| Tsien, 2022 [27] | HF-SRT | All | 54 (4-412) | NR | NR | 0% |
| Bergman, 2020 [33] | HF-SRT | All | NR | NR | 140 | 0% |
| Schnell, 2015 [35] | Conventional RT | All | reRT + concomitant Bev= 136.6  reRT + concomitant/maintenance Bev= 105.8 | 19.2 | 96 | 0% |
| Baehr, 2020 [40] | Conventional RT | Some | 120.5 (25-580) | 10 (3-54) | 99.4 | 0% |
| Hasan, 2015 [41] | HF-SRT | Some | NR | NR | 102.88- 126.66 | 0% |
| Yazici, 2014 [36] | HF-SRT | Some | NR | 15 (5-45) | 120 | 2.70% |
| Shen, 2018 [47] | Conventional RT | Some | NR | 27.6 (4.8-214.2) | 101.4 | 3.40% |
| Conti, 2012 [42] | HF-SRT | None | reRT + syst; Mean PTV 13.8 ± 8.3  reRT; Mean PTV 15.1 ± 8.2 | NR | 120 | 4.30% |
| Chan, 2020 [50] | HF-SRT | Some | 145.3 (10.6–432.8) | NR | 97.89 | 6% (reRT- 66.7%, reRT + Bev- 0%) |
| Eberle, 2020 [44] | HF-SRT (carbon-ion) | NR | NR | 10 (3-154) | 116.25 | 6.70% |
| Fleischmann, 2019 [18] | Conventional RT | Some | reRT; 122.46 (43.39–293.51)  reRT + Bev; 117.45 (22.55–385.5) | reRT= 18 (5–182)  reRT + Bev= 17 (4–265) | 96 | 6.8% (reRT- 13.5%, reRT + Bev- 4.8%) |
| Yasuda, 2018 [34] | HF-SRT | All | 33.9 (2.2-305.7) | 18.7 (1.3-438) | 144 | 6.9% (Bev administered adjuvantly-  RN resolved post Bev administration) |
| Hundsberger, 2013 [51] | HF-SRT | Some | 190 (47–373) | 40.9(6.1-387.9) | 108.46 | 7.1% (reRT- 25.0%, reRT + Bev- 0%) |
| Youland, 2018 [54] | HF-SRT | Some | 49 (3–265) | NR | 108.13 | 8.3% (reRT- 19.0%, reRT + Bev- 0%) |
| Scartoni, 2020 [46] | Conventional RT (proton) | None | 118 | 21.3 (5-96) | 96 | 9.10% |
| Cuneo, 2012 [53] | SRS | Some | 4.8 | NR | 123.75 | 9.5% (reRT- 19.0%, reRT + Bev- 4.7%) |
| Miwa, 2014 [38] | HF-SRT | None | Mean PTV 27.4 ± 24.1 (3.4 - 102.9) | NR | 120 | 9.50% |

Abbreviations: HF-SRT, Hypofractionated Stereotactic Radiotherapy; RT, Radiotherapy; SRS, Stereotactic Radiosurgery; reRT, reirradiation; Bev, Bevacizumab; PTV, Planning Target Volume; RN, radionecrosis; cc, cubic centimetre; NR, not reported
